# The 2.1 Ga Old Francevillian Biota: Biogenicity, Taphonomy and Biodiversity

**DOI:** 10.1371/journal.pone.0099438

**Published:** 2014-06-25

**Authors:** Abderrazak El Albani, Stefan Bengtson, Donald E. Canfield, Armelle Riboulleau, Claire Rollion Bard, Roberto Macchiarelli, Lauriss Ngombi Pemba, Emma Hammarlund, Alain Meunier, Idalina Moubiya Mouele, Karim Benzerara, Sylvain Bernard, Philippe Boulvais, Marc Chaussidon, Christian Cesari, Claude Fontaine, Ernest Chi-Fru, Juan Manuel Garcia Ruiz, François Gauthier-Lafaye, Arnaud Mazurier, Anne Catherine Pierson-Wickmann, Olivier Rouxel, Alain Trentesaux, Marco Vecoli, Gerard J. M. Versteegh, Lee White, Martin Whitehouse, Andrey Bekker

**Affiliations:** 1 Institut IC2MP, UMR 7285 CNRS-INSU, Université de Poitiers, Poitiers, France; 2 Department of Palaeozoology and Nordic Center for Earth Evolution, Swedish Museum of Natural History, Stockholm, Sweden; 3 Nordic Center for Earth Evolution, Odense M, Denmark; 4 Laboratoire Géosystèmes, UMR 8217 CNRS, Université Lille 1, Villeneuve d'Ascq, France; 5 Centre de Recherches Pétrographiques et Géochimiques, CNRS UMR 7358, Vandoeuvre-lès-Nancy, France; 6 Département Géosciences, Université de Poitiers, Poitiers, France; 7 Département de Préhistoire, UMR 7194 CNRS, Muséum National d'Histoire Naturelle, Paris, France; 8 IMPMC, Sorbonne Universités, UMR 7590, MNHN - UPMC - IRD UMR 206, Paris, France; 9 IMPMC, Sorbonne Universités, UMR 7590, MNHN - UPMC - IRD UMR 206, Paris, France; 10 Département Géosciences, UMR 6118, Université de Rennes, Rennes, France; 11 Stockholm University, Department of Geological Sciences, SE-106 91, Stockhom, Sweden; 12 Instituto Andaluz de Ciencias de la Tierra, CSIC-Universidad de Granada, 18100 Armilla, Granada, Spain; 13 Laboratoire d'Hydrologie et de Géochimie de Strasbourg, UMR 7517 CNRS, Strasbourg, France; 14 Société Etudes Recherches Matériaux, CRI Biopole, Poitiers, France; 15 IFREMER, Department of Physical Resources and Deep-Sea Ecosystems Technopôle Brest-Iroise, Plouzané, France; 16 Marum Center for Marine Environmental Sciences, Bremen, Germany; 17 Agence Nationale des Parcs Nationaux, BP. 20379, Libreville, Gabon; 18 Laboratory for Isotope Geology and Nordic Center for Earth Evolution, Swedish Museum of Natural History, Stockholm, Sweden; 19 Department of Earth Sciences, University of California, Riverside, Riverside, California, United States of America; University of Florence, Italy

## Abstract

The Paleoproterozoic Era witnessed crucial steps in the evolution of Earth's surface environments following the first appreciable rise of free atmospheric oxygen concentrations ∼2.3 to 2.1 Ga ago, and concomitant shallow ocean oxygenation. While most sedimentary successions deposited during this time interval have experienced thermal overprinting from burial diagenesis and metamorphism, the ca. 2.1 Ga black shales of the Francevillian B Formation (FB2) cropping out in southeastern Gabon have not. The Francevillian Formation contains centimeter-sized structures interpreted as organized and spatially discrete populations of colonial organisms living in an oxygenated marine ecosystem. Here, new material from the FB2 black shales is presented and analyzed to further explore its biogenicity and taphonomy. Our extended record comprises variably sized, shaped, and structured pyritized macrofossils of lobate, elongated, and rod-shaped morphologies as well as abundant non-pyritized disk-shaped macrofossils and organic-walled acritarchs. Combined microtomography, geochemistry, and sedimentary analysis suggest a biota fossilized during early diagenesis. The emergence of this biota follows a rise in atmospheric oxygen, which is consistent with the idea that surface oxygenation allowed the evolution and ecological expansion of complex megascopic life.

## Introduction

Reports of Paleoproterozoic macrofossils tend to be controversial, and considerable uncertainty persists about the nature of such remains. The report of centimeter-sized pyritized fossils in the ca. 2.1 Ga Francevillian black shales of Gabon [1] portended a new window on the history of macroscopic multicellular life. Their putative biological origin was investigated with the help of non-invasive structural investigations in combination with Δ^34^S analysis elucidating the process of pyritization. The study concluded that the objects meet commonly accepted criteria of biogenicity [2] and thus are likely to be fossils. The fossils were interpreted to represent organized and spatially discrete populations of colonial organisms, exemplifying a possible pathway toward the emergence of multicellular macroorganisms.

Multicellularity has arisen a multitude of times in prokaryotes and eukaryotes [3], [4]. It has a long geological history [5] and most likely occurred also in numerous lineages not represented in today's biota. The concept comprises a wide spread of phenomena, from cooperation between cells in a colony to highly organized and genetically regulated cell and tissue differentiation within complex bodies. The evolutionary pathways from simple coloniality to complex multicellularity are probably diverse, and involve various issues of cell–cell recognition, competition, co-operation, and adhesion. A fundamental distinction is between divisional (cells staying together after division) and aggreagational (individual cells coming together during part of their life cycle) types of multicellularity; they have different evolutionary origins [6], [7], and the aggregational type seems confined to terrestrial environments [3].

Multicellularity does not necessarily imply large body size, but because of the limitations of diffusion rates and streaming in cytoplasm, non-microscopic body size requires multicellularity (or syncytiality, multiple nuclei within a connected cytoplasm, which is functionally equivalent to multicellularity and sometimes interchangeable with it) [8]. The purported significance of the Gabon fossils is thus not multicellularity as such but the evidence they provide for a first appearance in the fossil record of macroscopic individuality [1].

In extensive investigation of the FB2 Subunit outcropping near Franceville, Gabon, we have now identified at least forty-five fossiliferous black-shale levels and collected more than 400 specimens, including diverse types not represented in the originally described assemblage. We are aware of the potentially confounding effects of some sedimentary (e.g., the binding of sediments by microbial mats) and diagenetic (e.g., the growth of concretions) processes that may produce macroscopic structures. The need to test mode of formation and origin is, therefore, critical. Accordingly, to evaluate further the spatial and chemical relationships of the structures to the surrounding rock, to elucidate their taphonomic history, and to assess their outer and inner structural variation and related biological diversity, we have submitted the newly available assemblage to detailed geochemical (δ^34^S) and morphological–structural (including microtomography) investigations.

## Geological, geochronological and sedimentary setting

**Figure 1:**
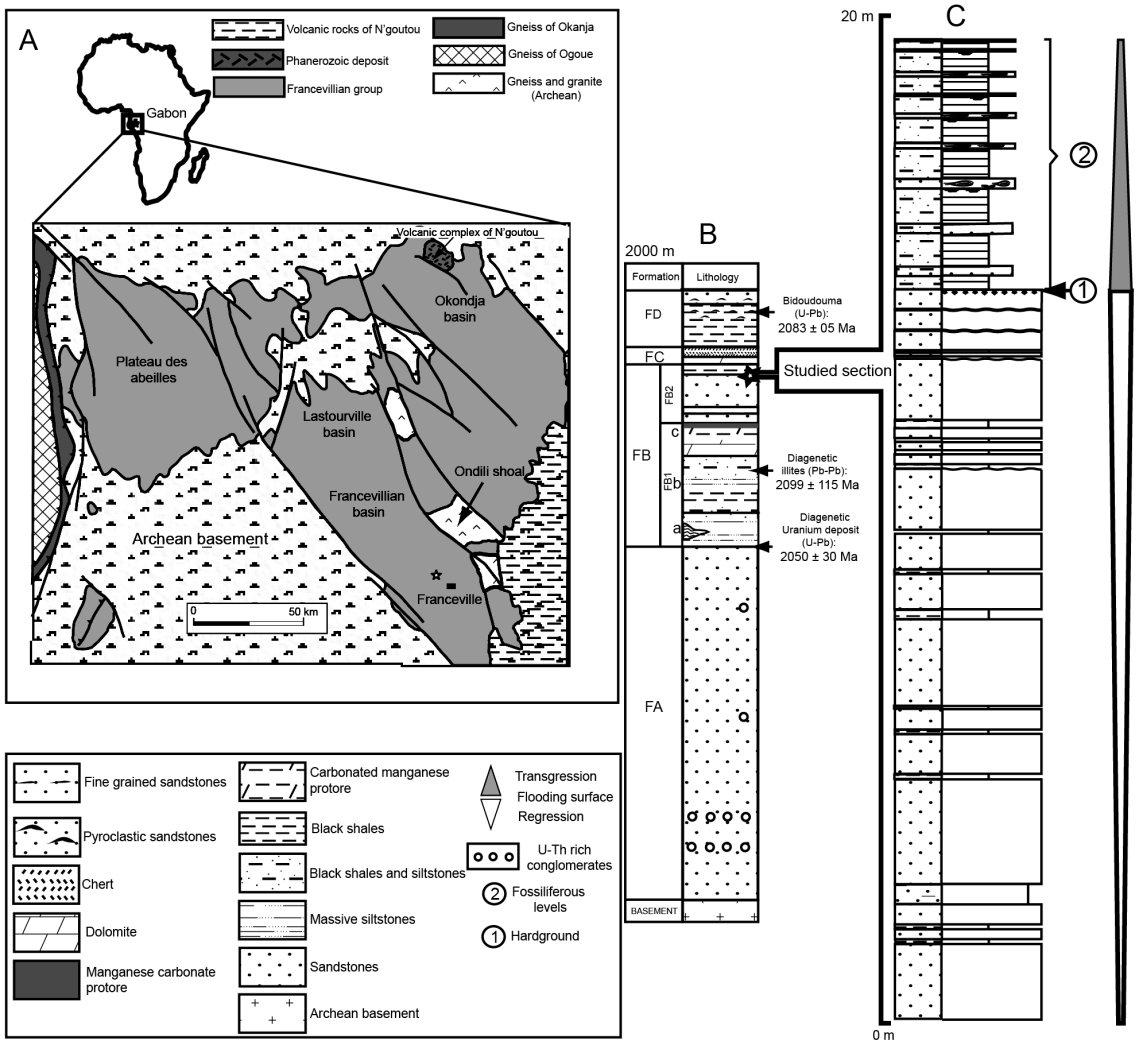
Geological map of the Francevillian basin and lithostratigraphy of the Paleoproterozoic Francevillian Series. (A) The location of the fossiliferous quarry is indicated by a star. (B) The Francevillian Series consists of four formations (FA to FD). The star indicates the FB2 Subunit. (C) Detailed lithology of the FB2 Subunit in the fossiliferous quarry.

The Francevillian basin consists of 35,000 km^2^ of unmetamorphosed sedimentary rocks, with no indication of hydrothermal influence [9]–[11], deposited during the Paleoproterozoic Eon in an epicontinental setting in what is now the Republic of Gabon, western equatorial Africa (Figure 1A). The sediment package is between 1000 and 2500 m thick and is subdivided into four lithostratigraphic units, FA to FD, which rest unconformably on Archean basement rocks [12] (Figure 1B, C). The FA unit consists of mainly fluviatile and deltaic sandstones. At the top, it contains uranium enrichments and hosts the well-known Oklo nuclear reactors [13]. The FB unit consists of marine sediments deposited mainly below storm wave base. Because of its diverse lithological composition, the FB unit is further divided into the FB1 (a, b, and c) and FB2 (a and b) subunits. The FB1a and FB1b subunits consist of interlayered shales, sandstones, and conglomerates, fining upwards to predominantly shales at the top. The FB1c subunit mainly consists of shales, but it also contains a thin iron formation overlain by black shales and a thick interval rich in manganese (Mn). The FB2a subunit consists of sandstone beds deposited in channels near the fair-weather wave base. These are sharply overlain by the FB2b subunit including finely-laminated black shales interbedded with thin siltstone layers deposited by waning storm surges. The previously reported large colonial organisms [1], and the new specimens presented here were collected from the FB2b black shales. In the fossiliferous quarry, the FB2b black shales are 5 m thick. The overlying FC Unit is dominated by dolomites and stromatolitic cherts, indicating shallow-water conditions. Stromatolites are found on topographic highs at the base of the FC unit [14]. The FD unit corresponds to black shales deposited during a transgressive phase.

Diagenetic illites from the top of the FB1b subunit yielded a Sm-Nd age of 2099±115 Ma [15], while a more precise U-Pb zircon age of 2083±6 Ma was reported from a welded tuff near the top of the FD unit [16]. In addition, a Rb-Sr whole-rock age of 2143±143 Ma has been reported for coarse-grained syenites of the N'Goutou alkaline complex, the emplacement of which is believed to be contemporaneous with the deposition of the base of the FB1 subunit [17]. Overall, the large portion of the Francevillian Series was deposited during the ca. 2.22-2.1 Ga Lomagundi carbon isotope excursion [18]. The preservation of randomly ordered smectite-rich illite/smectite mixed layer minerals (R0-type) demonstrates unusually slow mineral transformation and only a moderate degree of diagenesis, which is remarkable, considering the Paleoproterozoic age of the sedimentary succession [11].

The Francevillian fossiliferous black shales were deposited from an oxygenated water column in a quiet, low-energy marine environment [1], [18]. They are interbedded with thin sandstone layers lacking macrofossils. Lithofacies analysis indicates the absence of bedding-parallel microbial mats throughout the entire fossiliferous sequence. The macrofossils are distributed without significant overlap on the black-shale bedding planes. Fine laminae, prevailing in the enclosing shales, surround the specimens, indicating that the structures were in place before burial compaction [1]. Most specimens are completely to partially pyritized, yet some are only represented by impressions. Others are coated with iron oxides from pyrite oxidation. Both moulds and impressions are commonly preserved [1].

## Methods

High-resolution micro-computed X-ray tomography (micro-CT) of the macrofossils was run on a X8050-16 Viscom AG. Reconstructions were done using DigiXCT v.3.0 (Digisens) 64-bit version running on a Carry Systems workstation with 2 processors Intel Xeon 6 core 2.66 GHz, with 24 GB of DDR3 1333Mhz and 3 NVIDIA graphic cards (Quadro 6000 and 2 Tesla C2070). Virtual sections and 3D rendering were performed with Avizo Fire v.7.0 (VSG, Visualization Sciences Group).

Sulfur isotopes (^34^S to ^32^S ratios) were measured by Secondary Ion Mass Spectrometry (SIMS) using Cameca IMS1270 and 1280 instruments at CRPG (Nancy, France) and at the NordSIM facility (Stockholm, Sweden). The SIMS method for analysis of sulfur isotopes was described in detail in [19] and [20]. The sulfur isotopic compositions were measured using a 20 µm Cs^+^ primary beam of ≈2–5 nA. Sulfur isotopes were measured in multicollector mode using two off-axis Faraday cups (L2 and H1). The gains of the Faraday cups were intercalibrated at the beginning of the analytical session and the offsets were determined before each analysis during the pre-sputtering (300 s). Typical ion intensities of 3×10^9^ counts per second (cps) were obtained on ^32^S^−^, so that an internal error better than ±0.1‰ could be reached. Instrumental mass fractionation and external reproducibility were determined by multiple measurements of the in-house reference material Pyr3B (δ^34^S = +1.41‰) at CRPG, and Ruttan pyrite (δ^34^S = +1.2‰) and Balmat pyrite (δ^34^S = +15.1‰) at NordSIM. The external reproducibility ranged between 0.05‰ and 0.40‰ (1 σ) depending on the analytical session.

For palynology, about 25 g of each sample (*n* = 24) were macerated with HF/HCl followed by settling and decanting. Part of the residue was mounted on microscope slides. Single microfossils were handpicked under an inverted microscope by using a micropipette, then deposited uncoated on an aluminum stub and imaged by backscattered electron microscopy with a Jeol Environmental Scanning Electron Microscope (ESEM). Further analyses (FTIR, Raman, FIB, TEM, STXM, and Ultramicrotomy) of single palynomorphs are described below.

Scanning transmission electron microscopy (STEM) observations were performed using the high-angle annular dark field mode (HAADF) and a probe size of 1 nm. In this mode, brighter areas correspond to regions with higher atomic numbers. Energy dispersive x-ray spectrometry (EDXS) mapping was performed using the STEM mode. For Focused Ion beam (FIB) sectioning, an ultrathin electron-transparent foil was prepared by focused ion beam (FIB) milling using a FEI strata Dual beam at Lille University. FIB foils were lifted up and welded on one side onto a copper TEM grid *in situ* before final polishing. The FIB foils was studied at IMPMC (Paris, France) by transmission electron microscopy (TEM) using a JEOL 2100F (FEG) operated at 200 kV and equipped with a field emission gun, a high-resolution UHR pole piece, and a Gatan energy filter GIF 200. Scanning transmission x-ray microscopy (STXM) and x-ray absorption near-edge structure spectroscopy (XANES) analyses were carried out on the FIB ultrathin section at the carbon K-edge (C K-edge) at the Advanced Light Source (Lawrence Berkeley National Laboratory, Berkeley, USA) on the Polymer STXM 5.3.2.2 beamline, providing theoretical energy resolution better than 0.1 eV and a spatial resolution better than 25 nm. Raman spectra were collected on isolated microfossils. Spectra were recorded from nearly twelve different points in each sample to ensure the representative nature of the spectra, by means of a Renishaw inVia Reflex Raman Microprobe using a Peltier-cooled charge. FTIR Spectra were recorded on single microfossils using an IR source and Cesium Iodine (CsI) beamsplitter from a Nicolet 6700 FT-IR spectrometer coupled with a Thermo Scientific Nicolet Continuum FT-IR microscope equipped with a Mercury Cadmium Telluride (MCT) detector cooled with liquid nitrogen. Analyses were performed in transmission mode in the Middle Infrared (MIR) domain between 4000 and 400 cm^−1^. For ultramicrotomy preparation, single microfossils were embedded in agar and dehydrated in ethanol solution. Samples were polymerized at 60°C for 12 h. Ultra-thin sections (50–60 nm-thick) were cut from the resin blocks with a diamond knife.

Illustrated specimens are deposited at the University of Poitiers and the Swedish Museum of Natural History, Stockholm (SMNH numbers). Here below the references:

(G-FB2-s-615), (G-FB2-s-614), (G-FB2-s-608), (G-FB2-s-606), (G-FB2-s-605), (G-FB2-s-604), (G-FB2-s-601), (G-FB2-s-600), (G-FB2-s-593), (G-FB2-s-589), (G-FB2-s-586), (G-FB2-s-82), (G-FB2-s-259), (G-FB2-s-71), (G-FB2-s-576), (G-FB2-s-575), (G-FB2-s-573), (G-FB2-s-423) (G-FB2-s-123), (G-FB2-s-49a), (G-FB2-s-118), (G-FB2-s-148), (G-FB2-s-160).

Details of permits: the permits are provided by the Centre National Pour la Recherche Scientifique et technique du Gabon (CENAREST). Permit number: GA/488.

## Results

### Macrofossils

The specimens described here have been excavated from 45 horizons within the 5 m-thick section of the FB2b black shales. There are three main categories of putative macrofossils: pyritized forms (Figures 2, 3, 4, 5, 6, Figures S1–S9 in File S1), including the lobate forms described earlier [1] and apparently related elongated forms, non-pyritized circular disks on the shale surfaces (Figures 7, 8A, B, C, E), and a rounded aggregate of disk-shaped or flattened globular subunits (Figure 8C, D, E, F).

**Figure 2:**
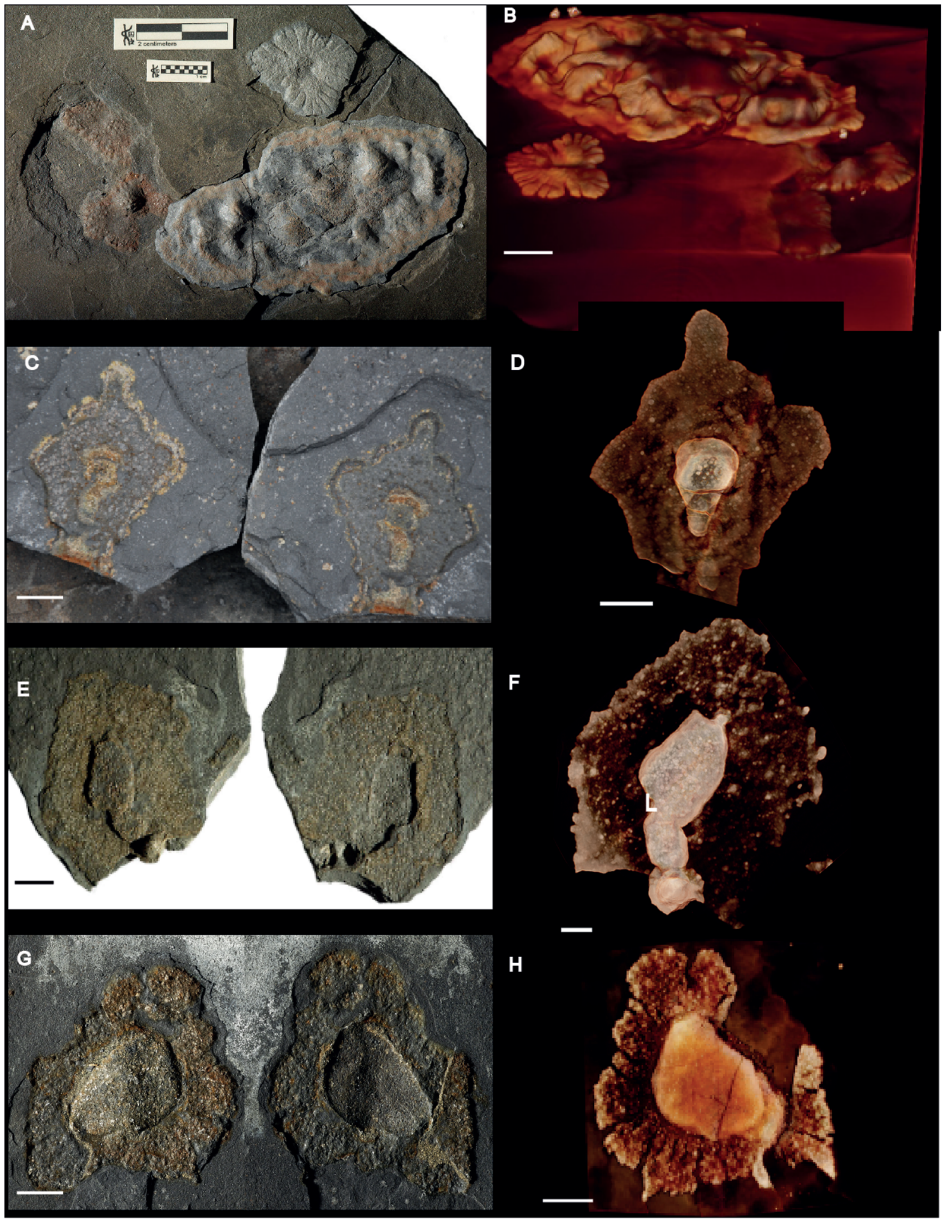
Pyritized macrofossil assemblage from the FB2 black shales of the Francevillian Series, Gabon. Photographs and micro-CT volume renderings in semi- or full transparency. Where both part and counterpart are shown, the left side of the pictures shows the stratigraphic surface viewed from above, impressions in the overlying black shale are on the right side. (A–H) Lobate forms showing sheet-like structure, radial fabric (A, B, G, H) and wrinkled appearance (A). Scale bars 1 cm.

**Figure 3:**
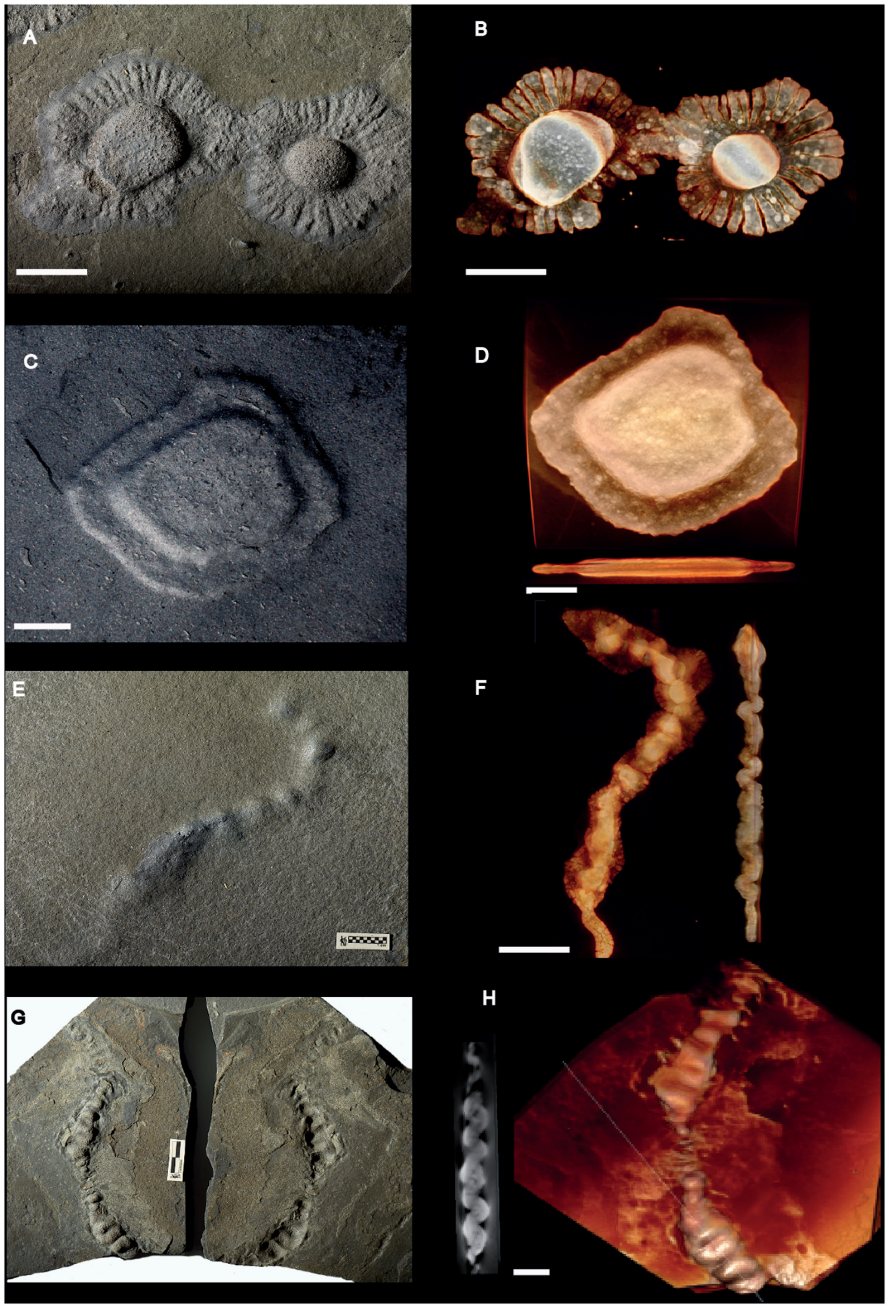
Pyritized macrofossil assemblage from the FB2 black shales of the Francevillian Series, Gabon. Photographs and micro-CT volume renderings in semi- or full transparency. (A–D) Lobate forms showing sheet-like structure and radial fabric (A, B). (E–H) Elongate forms showing sinuous shapes and tightly folded structure. In G, both part (positive epirelief, left) and counterpart are shown. Scale bars 1 cm.

**Figure 4:**
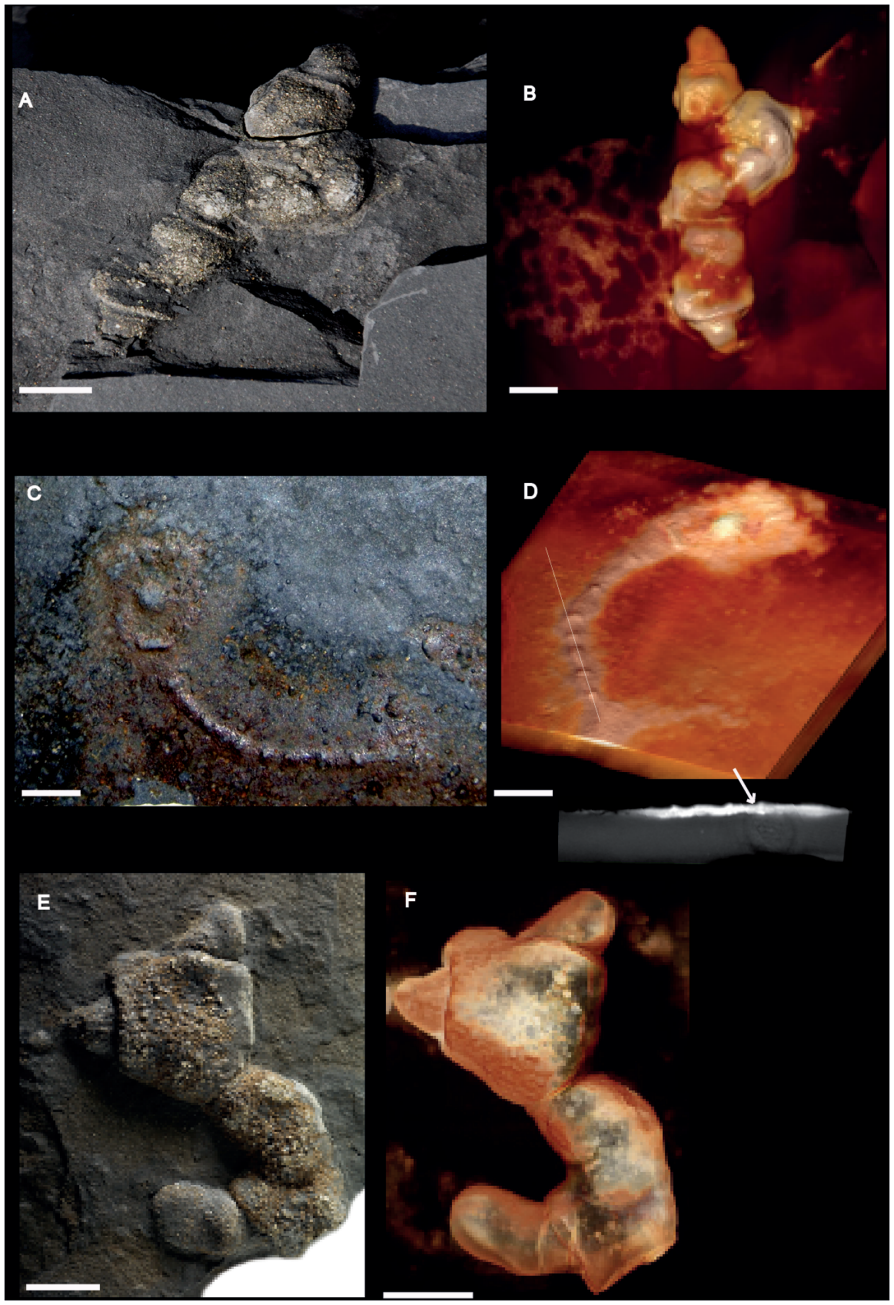
Pyritized macrofossil assemblage from the FB2 black shales of the Francevillian Series, Gabon. Photographs and micro-CT volume renderings in semi- or full transparency (A–F) Forms with partly or wholly elongated morphology. (A, B) Strongly pyritized specimen with traces of degraded sheet. (C, D) Specimen combining lobate and elongate morphology. (E, F) Strongly pyritized specimen with probably little original morphology preserved. Scale bars 1 cm.

**Figure 5:**
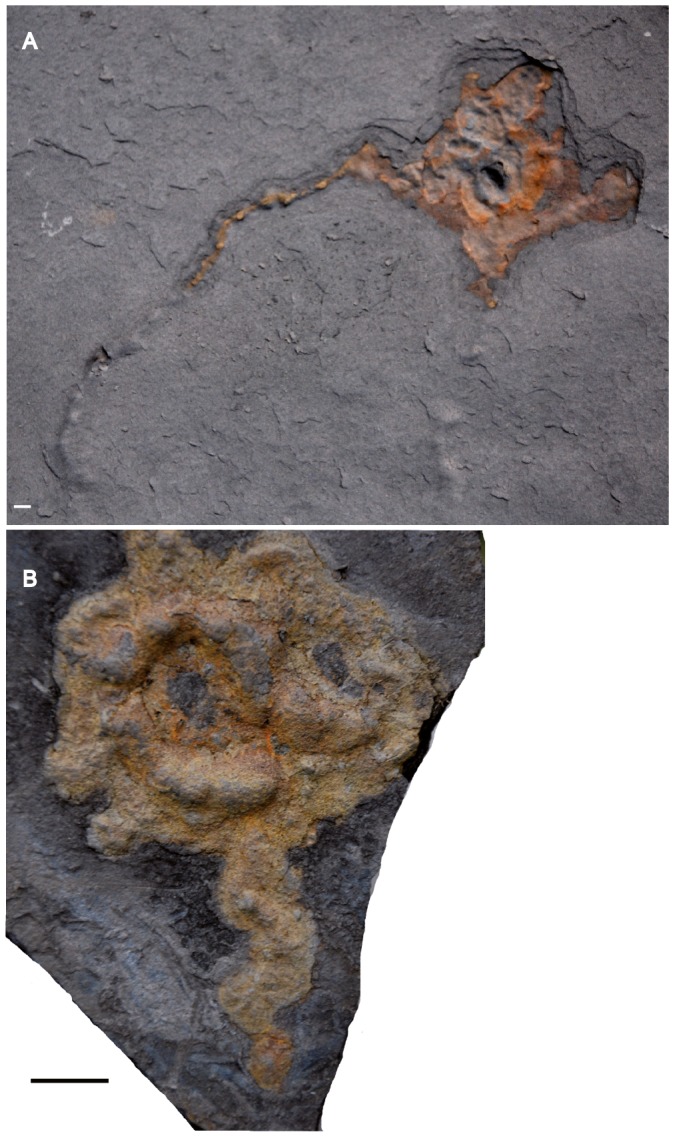
Photographs of pyritized macrofossil assemblage from the FB2 black shales of the Francevillian Series, Gabon. The size and fragility of these specimens did not permit the use of micro-CT observation. (A, B) Forms combining lobate and elongated, partially sinuous, morphology. Scale bars 1 cm.

**Figure 6:**
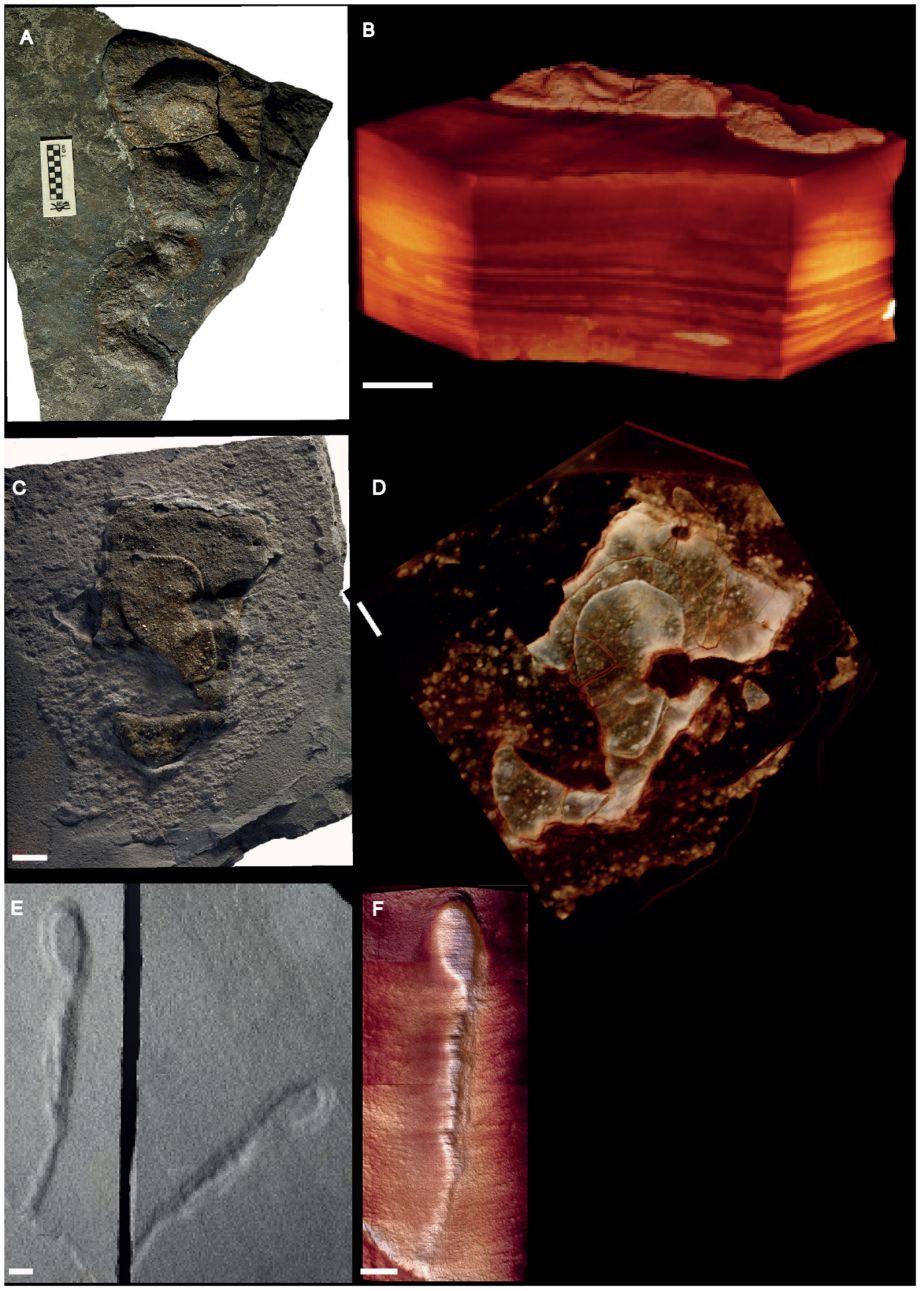
Pyritized macrofossil assemblage from the FB2 black shales of the Francevillian Series, Gabon. Photographs and micro-CT volume rendering in semi- or full transparency. (A–F) Forms combining lobage and elongate morphology. The “knobs” in the elongate middle portion are shown in specimen (E, F). Scale bars 1 cm.

**Figure 7:**
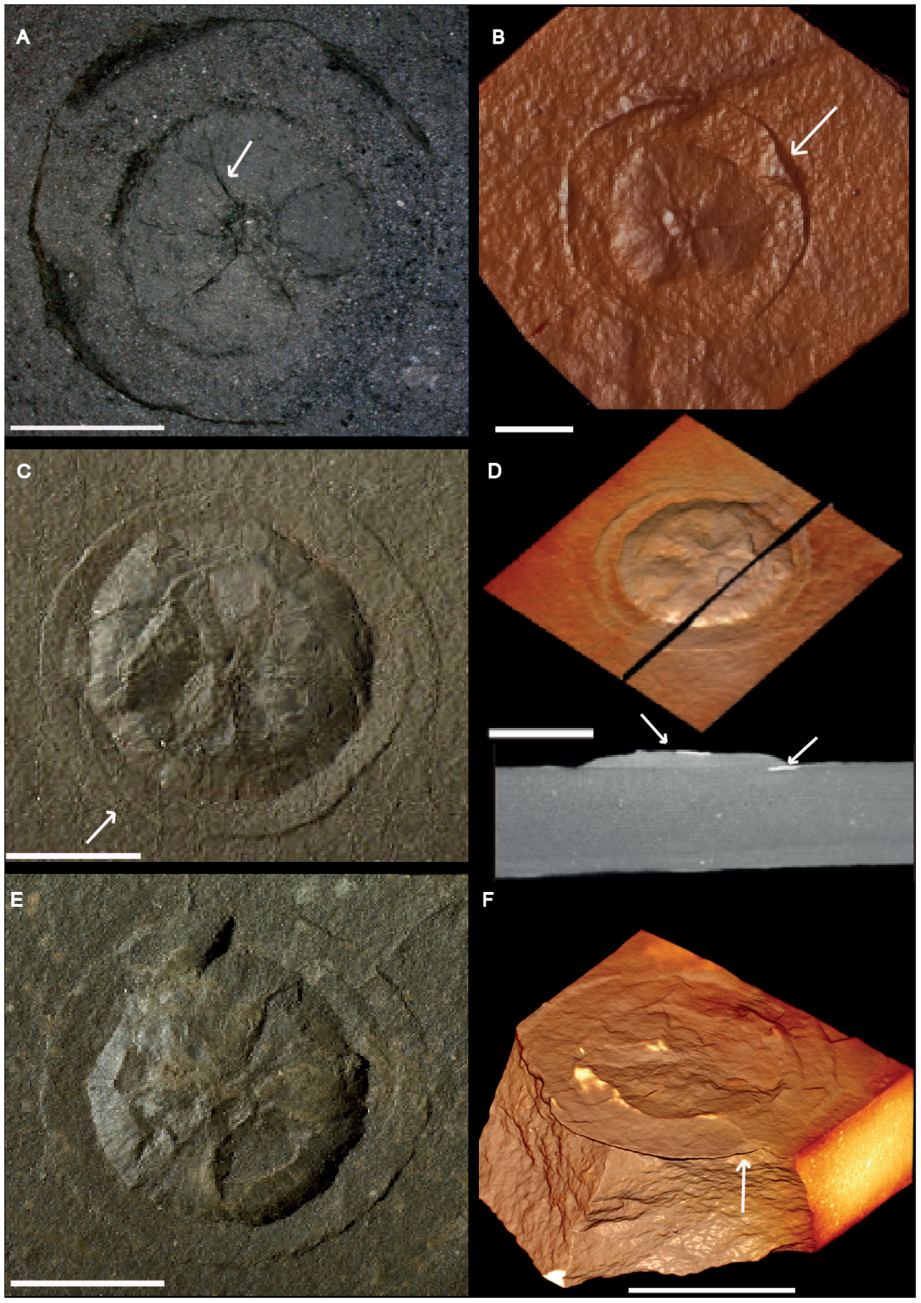
Macrofossil assemblage from the FB2 black shales of Gabon. Photographs and micro-CT volume rendering in semi- or full transparency and different projections show the disparity of forms from the FB2 Subunit and their diverse inner structural organization. Spatial resolution varies from 30 to 115 µm^3^. (A–F) Non-pyritized to weakly pyritized disks with a radially striated core encircled (arrow) by a flange-like outer part. Virtual section (bottom) in D shows the sharp contact between the specimen and the laminae of the surrounding black shale. Arrows show a few remains of pyrite. Scale bars 1 cm.

**Figure 8:**
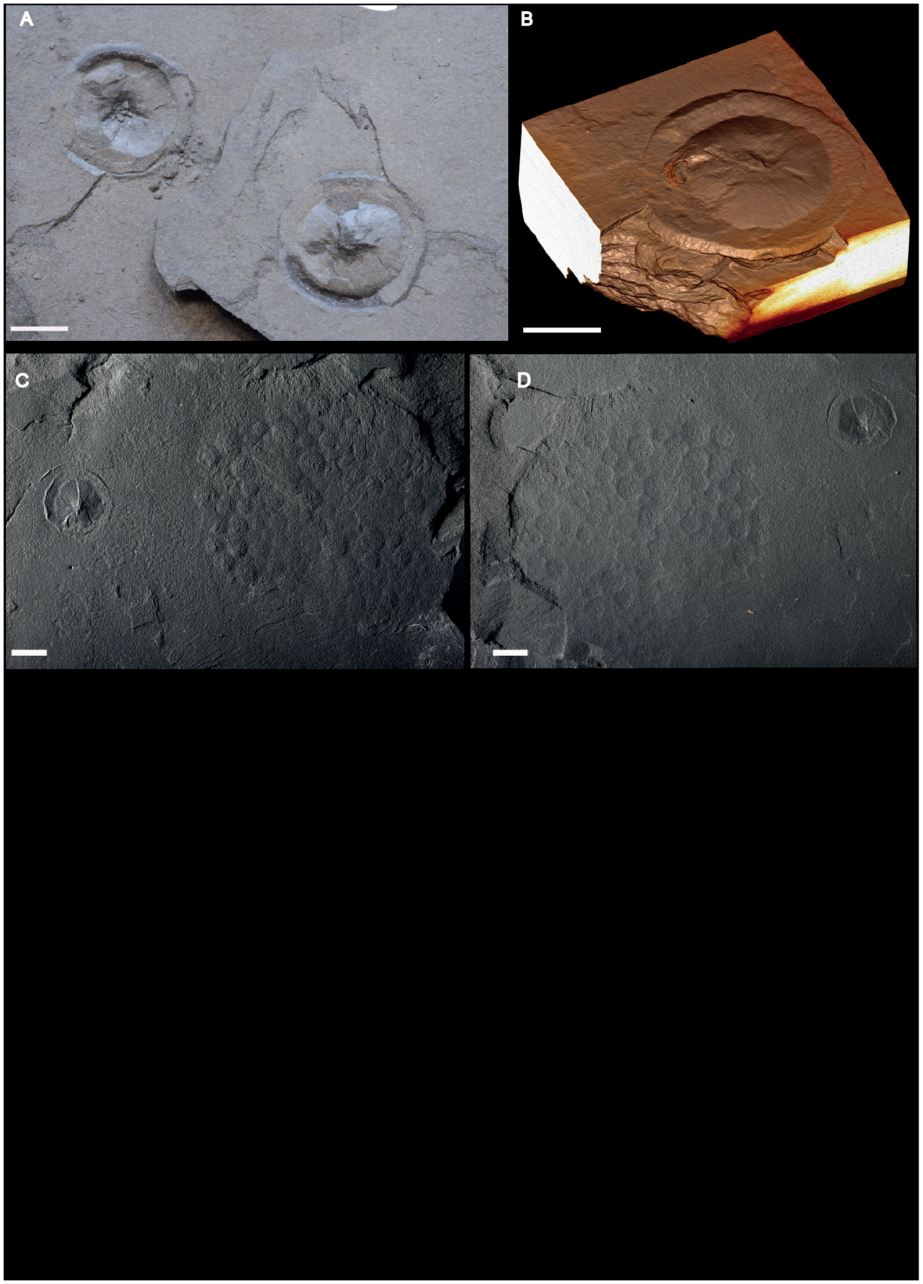
Macrofossil assemblage from the FB2 black shales of Gabon. Photographs and micro-CT volume renderings in semi- or full transparency (A, B, and C, left) Disks with radially striated core and flange-like outer part. (A) Part and counterpart of specimen. (B) Volume rendering in semi-transparency of the same specimen confirms the sharp contact between the specimen and host sediment. (C, D) Disk (left) and large circular aggregate showing slightly domed circular subunits. Scale bars 1 cm.

Fossiliferous levels may display more than one morphotype (Figure 8C), in particular, non-pyritized or weakly pyritized disks are associated with pyritized specimens. The abundance of fossils reaches occasionally more than 40 specimens per square meter. The lobate morphology is the most abundant of categories (∼40%), while elongated and disk-shaped specimens each amount to ∼30%.

All specimens originate from finely laminated black shale levels. Lithofacies analysis indicates quiet depositional conditions without evidence for reworking [1], [11]. The fossil abundance decreases towards the top of the section, where silty and sandy beds are more common. This suggests that higher hydrodynamic energy and shallower water depth were less favorable for fossil preservation.

#### Pyritized forms

The objects earlier reported by us [1] represent a group of lobate forms consisting of two distinct types of pyrite: a sheet-like structure preserved as euhedral 5–50 µm pyrite crystals dispersed in the shale matrix, and dense, coarsely crystalline pyrite, typically accumulating in the central parts of the specimen ([1]; see also under “Taphonomy and diagenesis of the pyritized fossils”). We refer to the former structure as the *sheet* and the latter as the *core*.

Lobate specimens range from nearly isodiametric to elongated and are variable in size (length: ∼7–170 mm; width: ∼5–70 mm; thickness: ∼1–10 mm). The sheets typically thin toward the periphery and are often folded in the central region, with a folding direction roughly perpendicular to the length axis ([1], Figure 4, two middle rows; Figure S2 in File S1). The sheets commonly have a permeating radial texture toward the outer edge (Figures 2A, top, 2B, bottom, 2G, H, 3A, B, Figures S2, S3 in File S1; and Figures 3 and 4 in [1]). This pattern is most pronounced on the periphery, but is sometimes expressed in the intermediate region between the central part and the periphery (Figure S2 in File S1 and Figure 4 bottom in [1]). The slits in the radiating texture are formed by pyrite-free regions [1]. Specimens with weakly expressed radial fabric (Figure 2G, H) indicate that the absence may reflect a difference in preservation (compare Figure 2G, H with 2E, F). Where the fabric is missing, the pyrite in the sheets tends to be more unevenly distributed, forming scattered granules in the shale (Figures 2F, 6D, Figures S2, S9 in File S1). Independent of the presence of folds or radial fabric, the lateral margin of the sheets is in most cases undulate or lobate (Figures 2A, top, 2B, bottom, 2C, D, G, H, 3A, B), but where the radial fabric is missing the outlines tend to be more irregular (Figures 2A, bottom, 2B, top, 2E, F, 6C, D).

The dense, coarsely crystalline core is in almost all cases distinctly demarcated from the sheet fabric. In CT scans it is seen as a bright lump in the center of the specimens (Figures 2D, F, H, 3B, Figures S2, S3, S7, S9 in File S1), and sections through the specimens also show the distinction between the core and the sheet fabric.

The new material contains elongate morphologies that at first glance appear very different from the lobate specimens so far described. Curved to sinuous strings roughly parallel to the shale laminae show a knobby texture, with rounded knobs 0.5–1.5 cm apart on the shale surface (Figure 3E, G). CT images, however, show this texture to be formed by tight transverse folds, similar to that seen in the sheet of several of the lobate specimens (Figure 3F, H). Furthermore, numerous elongate specimens show portions with flat sheets and peripheral radial fabric like the lobate ones (Figures 4C, D, 6A), again suggesting a likely link between these morphologies (compare with the elongated lobate forms shown in Figures 2–3 in [1]). Other elongate specimens consist of broader sheets connected to a narrow and elongated part (Figures 4C, D, 5, 6E, F) without visible radial fabric, but given their apparent poor preservation and the presence of such fabric in otherwise similar forms (e.g., Figure 6A, B) it is nevertheless likely that they are varieties of the same structure. Elongate specimens or parts commonly have an elliptic transverse cross-section (Figure S1 in File S1) and follow a curved to sinuous course (Figure 3E–H, 4, 5B, 6A, B).

A variety of both broader and narrower pyritized structures is represented by the specimens in Figures 3C, D and 6E, F, respectively. As in the forms described above, there is a central, more heavily pyritized portion, but it is flat rather than lump-shaped and seems to have formed in strict confinement by the shale laminae. It thus differs from the more lumpy core of the other specimens.

#### Non-pyritized circular disks

The circular disks are ∼8 to ∼40 mm in diameter (Figures 7, 8A, B) and are preserved in positive epirelief. They have a domed central part with a few coarse and numerous fine radial striations, and a well-demarcated flange-like outer part. A virtual section based on micro-CT (Figure 7D, bottom) shows that the body of this specimen is slightly more X-ray attenuating than the adjacent shale, with which it forms a sharp contrast. This is likely due to a higher content of disseminated pyrite; the specimen also has flakes of pyrite in the flange and central dome (light streaks in image).

#### Rounded aggregate

On the same bedding plane as some of the circular disks has been found a rounded aggregate, ∼80 mm in diameter, composed of a tight assemblage of slightly domed circular subunits, the diameter of which varies from ∼3 mm to ∼7 mm (Figure 8C, D). The subunits are preserved in positive epirelief. There is no evidence of textural or compositional difference between the material in the subunitis and that of the underlying shale.

#### Taphonomy and diagenesis of the pyritized fossils

Because the macroscopic fossil structures from the FB2 Subunit are mostly preserved in pyrite, their taphonomy and biogenicity were assessed through petrographic analysis (Figures 9, 10) and δ^34^S measurements (see Methods and Supporting Information) performed on nine specimens from this assemblage variable in morphostructure and dimensions (Figures 9, 11, Figures S1–S9 in File S1 and Table S1). For comparison, we also analyzed two types of pyrite concretions in Phanerozoic rocks (Figures 9, Figures S10, S11 in File S1).

**Figure 9:**
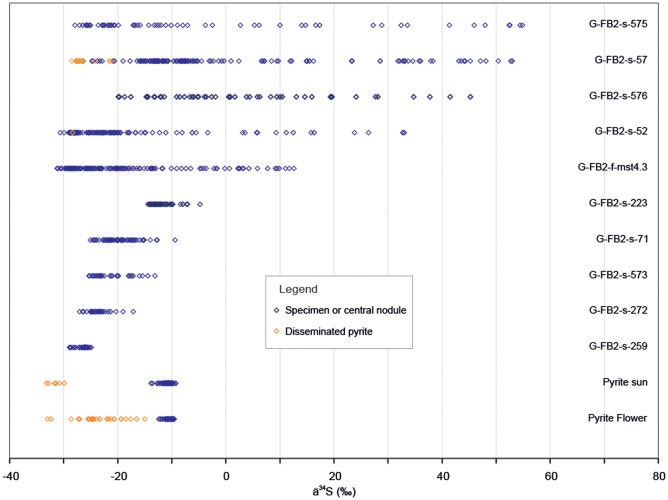
Results of δ^34^S isotopic analyses performed on two pyrite concretions (pyrite “sun” and pyrite “flower”, in blue) and on fossil specimens from Gabon (top, in blue) compared to those from their respective host sediments (orange). Host sediment values for the pyrite “flower” from [68] and [69].

**Figure 10:**
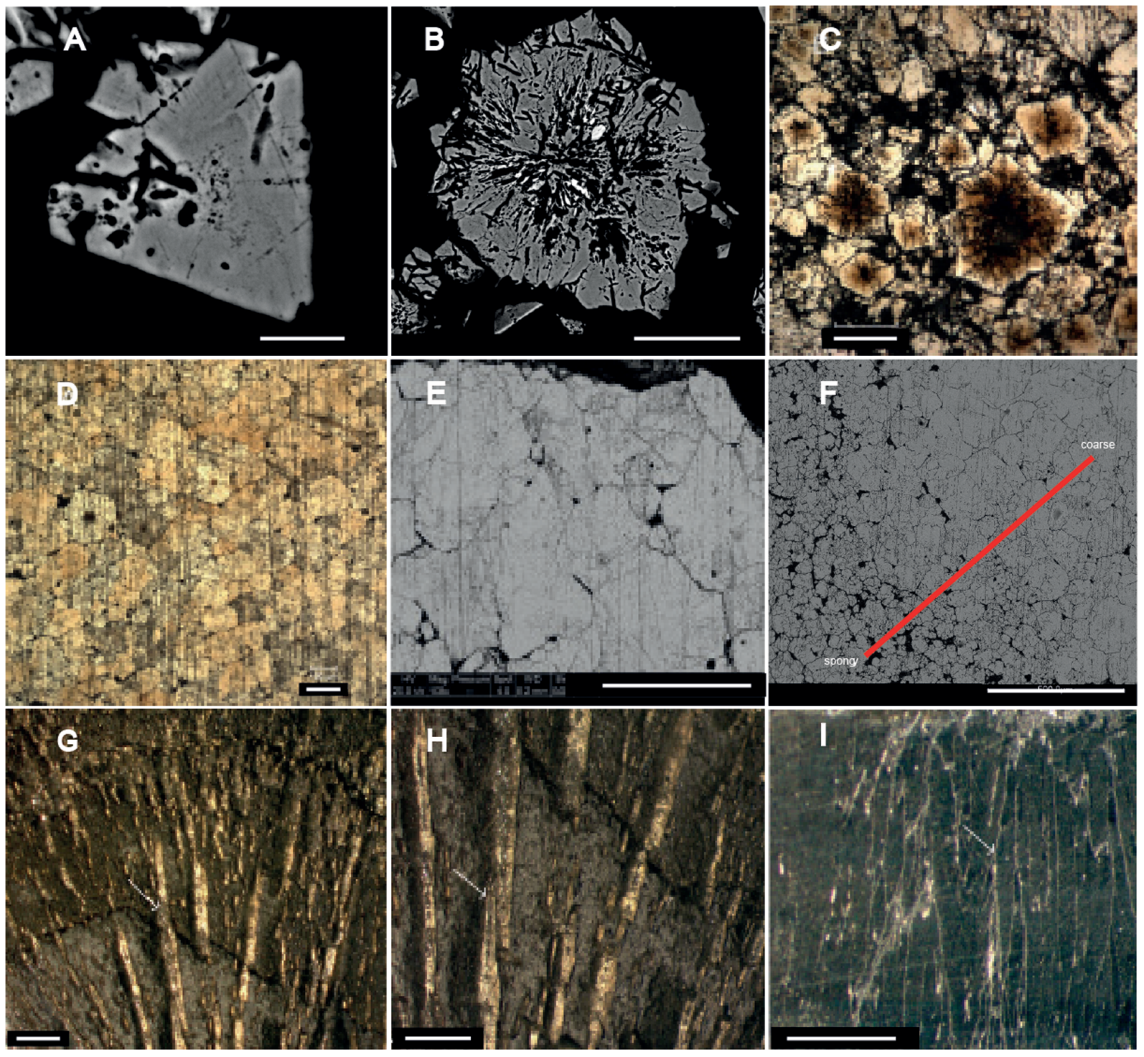
Petrography of pyrite crystals. (A) SEM-BSE image of an euhedral crystal after severe HNO_3_ etching. Note the spongy texture in the centre and the growth bands in the outer part. (B) SEM-BSE image of floriform pyrite after severe HNO_3_ etching. Note the radiating texture in the centre and the growth bands in the outer part. (C) Photomicrograph of the spongy pyrite after severe HNO_3_ etching. This pyrite contains both euhedral and floriform pyrites. Note the orange colour in the centre of euhedral and floriform pyrites. (D) Photomicrograph of coarse pyrite after severe HNO_3_ etching. Note small orange forms in the middle of some crystals. (E) SEM-BSE image of coarse pyrite after severe HNO_3_ etching. (F) SEM-BSE view of the transition from spongy (lower left corner) to coarse (upper right corner) pyrite. (G–I) Pyrite “sun” sample (SMNH X4450) under reflected, plane-polarized light, highlighting the growth texture of pyrite. (G, H) Surface of the pyrite “sun” showing apparent growth bands (underlined in red) and elongated radiating crystals (arrow) centrifugally developed perpendicular to the growth bands. (H) Close-up view of G showing the relationships between radial crystals (arrow) and apparent growth bands. (I) Section parallel to the plane of the pyrite “sun” showing a centrifugal arrangement of coarse acicular crystals (arrow). Scale bars 10 µm (A), 50 µm (B, C), 100 µm (D, I), 500 µm (E, F), and 5 mm (G, H).

**Figure 11:**
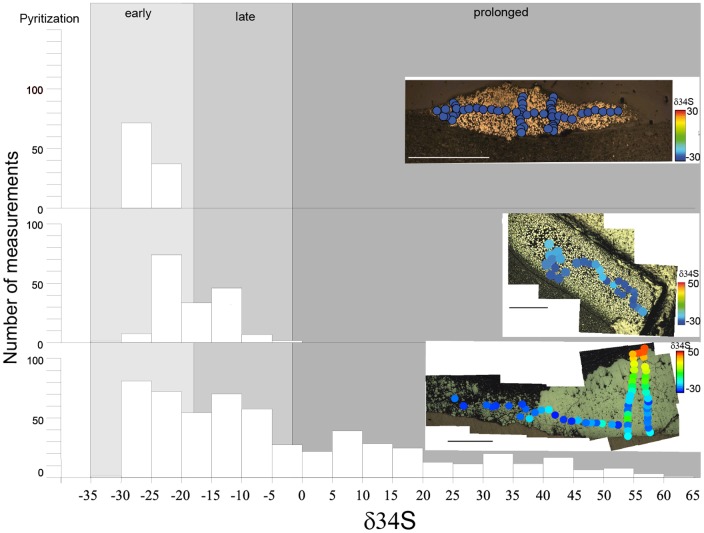
Results of δ^34^S analysis performed by secondary ion mass-spectrometry (SIMS) on selected specimens. Three different isotope patterns are distinguished and related to the stage of pyrite formation during diagenesis. Each case is illustrated by one histogram of δ^34^S data and the color data plot of a representative specimen. Early growth of pyrite (example from Figure S1 in File S1): pyritization was rapidly completed during early diagenesis. Late growth of pyrite (example from Figure S4 in File S1): pyritization continued with burial through early to somewhat later diagenesis. Prolonged growth of pyrite (example from Figure S9 in File S1): pyritization continued episodically over a large range of burial depth during late stages of diagenesis. An atlas showing specimens and their δ^34^S data is available in Supporting Information.

Pyritization of organic matter typically occurs through carbon remineralization by sulfate reduction where dissolved ferrous iron is available, such as in sediment pore-waters [21], [22]. Pyritization during early diagenesis occurs if sulfide production is localized at the site of the decaying organic matter and the iron is supplied from the pore fluid. Pyritization can also occur during multiple stages of diagenesis, through pyrite dissolution and precipitation leading to, for example, radial pyrite nodule growth [23]. The pattern of isotopic compositions of sulfur within the pyrite can help to distinguish between these scenarios [22], [24].

Pyrite disseminated in the FB2 sediment is dominated by 1–10 µm euhedral crystals. In contrast, in the macroscopic specimens, four different pyrite textures were observed: (a) euhedral pyrite, (b) floriform pyrite, (c) spongy pyrite, and (d) coarse pyrite.

Euhedral crystals in the specimens are in the range of 5–50 µm (File S1, Figure S12 in [1]) and are therefore mostly larger than disseminated pyrite in the shale. They are either isolated or clustered. Detailed petrographic study indicates that the larger euhedral crystals correspond to overgrowths (Py1) on originally smaller (<10 µm) masses of anhedral pyrite (Py0; Figure 10A). Growth bands can be observed in the outer parts of the crystals, and after acid etching the central anhedral pyrite often shows a spongy texture (Figure 10A). This anhedral, weakly crystallized texture (Py0) could reflect rapid crystallization of iron sulfide [25].

Floriform pyrites have a diameter of 50–100 µm. They are also either isolated or clustered. They generally show a radiating texture (Figure 10B). The central part is anhedral and radiating (Py0), while the outer part shows euhedral features and growth bands similar to the Py1 of the smaller euhedral crystals. These textures also probably reflect relatively rapid crystallization.

Spongy pyrite corresponds to a mosaic of ∼100 µm subhedral to euhedral crystals more or less coalescing, with sediment trapped in between. The crystals mostly consist of abundant smaller euhedral crystals and floriform pyrites cemented by a later generation, Py2, of subhedral pyrite (Figure 10C). Coarse pyrite corresponds to anhedral to euhedral crystals larger than 100 µm, which sometimes have growth bands (Py3). These crystals are generally coalescing (Figure 10D, E), and occasionally include small euhedral crystals (Figure 10D). The transition from spongy pyrite to coarse pyrite is more or less gradual (Figure 10F) and mostly occurs from the centre to the border: the central part is dominated by spongy pyrite containing small euhedral crystals in abundance. The size of pyrite crystals increases outwards and progressively passes to coarse crystals. This texture typically reflects slow crystallisation [25].

In most specimens the dominant texture is small euhedral pyrite dispersed in the shale matrix. Spongy and coarse pyrites are only observed in the densely pyritized parts of specimens. Coarse pyrite mostly occurs in the outer parts of cores, but can also be present more centrally. Apart from the absence of framboids, these pyrite textures are very similar to the textures described in pyritized soft tissues of Phanerozoic fossils, e.g. ([26]–[29]).

Where small euhedral and floriform pyrites are largely dominant, the isotopic signal shows low variability (<5‰) in the specimen (see Figure 11), reflecting precipitation under open conditions, i.e., in contact with the seawater sulfate reservoir. Where spongy pyrite is present (i.e., Figures S2–S5 in File S1), the isotopic signal is more variable and the variability ranges between 5‰ and 20‰ (in Figure 11). This reflects precipitation under open to semi-closed conditions. No particular trend is generally observed, but where the spongy pyrite is dense, specimens can show an outward increase of the values (central core in Figure S3 in File S1, and locally in Figure S8 in File S1). In specimens where the size of pyrite crystals increases outwards, the spongy pyrite in the centre shows relatively low isotopic variability (Figures 10, 11), but the isotopic values rapidly increase outwards in parallel to the size of pyrite crystals (Figures S6–S9 in File S1, and Figure 5 in [1]). This behaviour was also observed in pyritized Phanerozoic fossils [26], [27] and indicates that pyrite precipitation began in open system and was followed by precipitation in closed conditions [22].

Based on both the characteristics of pyrite crystals and their isotopic composition, the precipitation of pyrite must have been rapid for most of the Gabonese specimens. Open-system conditions indicated by the sulfur isotopic composition of small euhedral and floriform pyrites (Py0, Py1) were achieved at the sediment-water interface or after very shallow burial. Overgrowths on these crystals (Py2) leading to the formation of the spongy texture were also relatively rapidly developed. From its isotopic composition indicating open to semi-closed conditions, the precipitation of this pyrite was achieved after shallow burial in the sediment, under conditions where sulfate consumption by bacterial activity was more rapid than its renewal by diffusion. The depth at which these conditions were reached highly depends on the rate of sulfate reduction, which in turn depends on the degradability of the organic matter and sediment permeability [22]. While the exact depth cannot be determined precisely, it should nonetheless not exceed a few centimetres in poorly permeable sediments. The slight outward increase in S isotope values of specimens, which is sometimes observed, reflects a Rayleigh distillation in the closed system, with the outer part being precipitated after isolation from the seawater sulfate reservoir.

The coarse pyrite (Py3) observed in the outward part of the pyritized specimens is characterized by most positive S isotope values and an outward increase in values. These features indicate that precipitation of this Py3 occurred after the other forms of pyrite, from the increasingly ^32^S-depleted sulfate remaining in the pore water, and therefore after burial in the sediment. Deformation of lamina around the specimens indicates that the precipitation of Py3 was before compaction and can therefore be considered as early to late diagenetic.

An alternative explanation for a relatively homogeneous signal observed in most specimens as well as for disseminated pyrite is recrystallisation and homogenisation of the isotopic signature. This phenomenon implies dissolution and re-precipitation of the different forms of pyrite present in the rock and needs significant fluid circulation in the rocks [30]. We have seen no indication for strong fluid circulation in the studied rocks and the high isotopic variability observed in some specimens indicates that homogenization did not occur. We therefore discard a metamorphic/hydrothermal origin for the isotopic signature.

Overall, the investigated sheets from the FB2 subunit, dominated by small euhedral and floriform pyrites, possess distinct and repeatable textural patterns as well as isotopic signals consistent with early diagenetic pyritization. Specimens lacking pyrite core display homogeneously negative δ^34^S values (−30 to −20‰; Figures 9, 11, Figure S1 in File S1). These values are similar to the composition of disseminated pyrite within the enclosing black shale (see G-FB2-s-57 in Figure 9) and are in the range of the lightest values for sedimentary pyrite deposited before the late Neoproterozoic [31]. The values in the Francevillian fossils imply early diagenetic formation of pyrite, where accessible sulfate allows large fractionations during microbial sulfate reduction [22], [32]. With regard to spongy and coarse pyrite accumulation, some specimens show homogenous negative values around −20 to −10‰ (Figures 11, Figures S3, S4 in File S1). In most cases, however, there is a clear distinction between less intensely pyritized sheets, which are highly ^34^S-depleted (−30 to −20‰), and central pyrite cores, which are considerably ^34^S-enriched (up to +55‰; Figures 11, Figures S7–S9 in File S1). In agreement with the original observations [1], there is commonly progressive ^34^S enrichment of the pyrite core from the spongy interior towards the more massive edges (Figures S6–S9 in File S1), indicating late-stage and even prolonged pyritization in sediment pore-waters with reduced concentrations of sulfate [22]. This provides strong support to our interpretation of the Francevillian pyritic structures as pseudomorphs of biological macroscopic remains that in some cases have been distorted by later concretionary pyrite growth.

#### Phanerozoic pyrite concretions

Accretionary growth and the morphologies associated with this type of growth are common to biological structures as well as to chemical and crystalline precipitates. A concretion that owes its shape to pyrite growth can be identified as such because of its centrifugal crystal texture building up the external shape [33], [34]. However, a similar shape can also result from the pyritization of a microbial halo that forms around a decaying body [35]. Microtextures, which are strongly dependent on biological, chemical, and physical parameters [27], [36] are therefore of great importance to ascertain the mode of formation of pyrite structures.

In order to compare with the Gabonese specimens, petrographic analyses were performed on two pyrite concretions: a Carboniferous pyrite “sun” and a Jurassic pyrite “flower” (cf. Figures 9, 10G–I, Figures S10, S11 in File S1and Table S1). These concretions are dominated by macroscopic elongate crystals, growing in fan-like shapes with intergrowth textures through the host sediment (Fig. 10G–I). The specimens show homogeneous isotopic composition through their cross-section and are more ^34^S-enriched compared to matrix pyrite (Figure 9 and Table S1). This indicates precipitation in a semi-closed to closed system during late diagenesis and later homogenization of S isotope values by fluid circulation and/or metamorphism [30], in contrast to the early diagenetic pyritization of the Francevillian macrofossils. In terms of crystal size and shape, the two types of Phanerozoic concretions also differ significantly from the Francevillian macrofossils, since they have a fully crystalline pyrite body (Figures S10, S11 in File S1) rather than disseminated pyrite grains in shale matrix (Figures S1–S9 in File S1). Their morphology is clearly determined by the growth of the crystals.

Seilacher [33] interpreted the inner, lumpy, and the outer “skirt” part of a pyrite “flower” to represent two generations of pyrite growth, pre- and post-compaction, respectively [33]. Though such an interpretation makes sense from a mechanistic point of view, it is not supported by the uniform sulfur-isotope signal, which is more consistent with a single event of formation. Seilacher's two-step model may still be valid, however, if the pyrite in the “skirt” resulted from remobilization of iron sulfide in the core under closed-system conditions. In any case, our textural and isotopic study clearly demonstrates that Francevillian macrofossils are different from pyrite “suns” and pyrite “flowers”, indicating that the Gabonese fossils experienced completely different processes of pyritization.

### Microfossils

**Figure 12:**
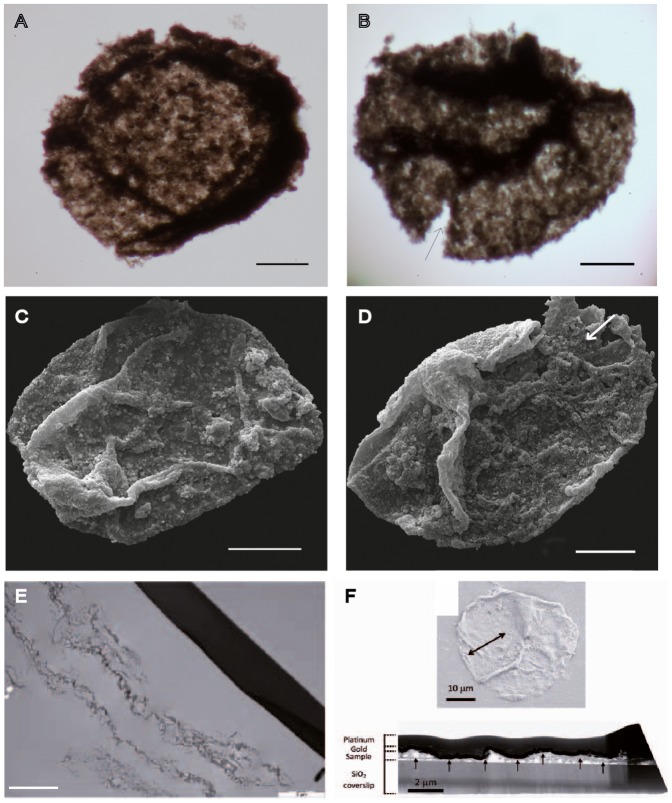
Spheroidal carbonaceous palynomorphs extracted from the host sediment by acid maceration. (A–D) Pictures obtained with a transmitted light microscope (A, B) and environmental SEM (C, D). Folding and wrinkling as well as granular and degraded textures of vesicle walls are likely taphonomic features. V-shaped cuts (B) and holes (D) (arrows) illustrate the vesicle wall structure. Scale bars 50 µm. (Extensive details, including Raman, STXM, FIB, TEM, and FTIR are available in Supporting Information.) (E) Ultramicrotomy section through the organic-wall of a single specimen. Scale bar 5 nm. (F) SEM image (top) of a specimen used to extract and FIB foil.Double arrowhead shows the location from where the FIB foil was extracted. Bright-field TEM image (bottom) of the FIB foil. The dark upper layer, which measures ∼800 nm in thickness, is the platinum strap deposited at the top of the gold-coated sample before FIB milling. Gold coating can be observed as a darker, ∼200 nm thick layer at the top of the specimen. The wall consists of a continuous carbonaceous film (arrows). It is mixed with various mineral particles. The sample lies over a glass coverslip. Scale bars 10 µm (top) and 2 µm (bottom).

In order to investigate the potential for microfossil preservation, 24 samples of siltstones and silty black shales from the FB2 Subunit were macerated for palynological investigation. The optical microscopic analysis of the organic residues revealed the presence of carbonaceous spheroidal microstructures 50–80 µm in diameter (Figure 12). These palynomorphs have highly granular wall texture and commonly show darker bands that appear to be thickenings or wrinkles on the original surface. This is confirmed by SEM observations (Figure 12C, D). V-shaped cuts or tears are commonly observed along the granular margins, demonstrating that these structures were originally cohesive. For one specimen, SEM revealed what appears a spongy microstructure on the inner side of the vesicle wall (Figure 12D). TEM observations showed that the specimen wall consists of a loosely attached complex of carbonaceous material (Figure 12E, F) in association with holes left by embedded mineral grains that were removed during acid treatment. Analysis of the FIB milled foil by scanning transmission x-ray microscopy (STXM) at the C K-edge (Figure S12 in File S2) confirms that the wall is composed of organic matter.

Several spectroscopic methods (XANES, FTIR, RAMAN; Figures S12–S16 in File S2) were used to characterize the organic matter and they point to a relatively aromatic material containing some carboxylic and aliphatic molecules. The relative aromaticity suggests that organic matter has experienced some thermal degradation.

The microstructures and the organic matter dispersed in the host sediment show similar RAMAN spectra (Figure S13 in File S2), suggesting that their carbonaceous material has a similar degree of maturity. These data corroborate the syngenicity of the studied microstructures within the host sediment.

The observed microstructures can be attributed to a group of organic-walled microfossils, which are increasingly recognized in macerations of Mesoarchaean [37] and Mesoproterozoic [38] siliciclastic sediments. The microfossils described from the ca. 3.2 Ga Moodies Group in South Africa and interpreted as evidence for a diverse ecosystem in the photic zone of marginal-marine siliciclastic environment are similar to those from the FB2 Subunit in that they also possess a continuous granular wall surrounding a central lumen, but are significantly larger (Figure 1d, e, f in [37]). Organic-walled microfossils classified as acritarchs have been described from many localities worldwide from younger (late Mesoproterozoic to Phanerozoic) sediments and are commonly interpreted as the encysted remains of eukaryotic algae [39]. Some of the similar, organic-walled microfossils in younger rocks are attributed to the eukaryotes because they possess complex wall structure (multi-layering, micro-sculpture) and complex vesicle morphologies indicative of cytoskeletal control of developmental morphology [40]. Spheroidal palynomorphs from the late Mesoproterozoic Eon (ca. 1.1 Ga) in West Africa include small forms (20–125 µm) with discrete, intact walls, interpreted as eukaryotes, and large forms (200–400 µm), comprising amorphous granular disks, interpreted as bacterial colonies associated with benthic microbial mats [38].

The currently accepted oldest non-spinose (sphaeromorphous) eukaryotes are from the ca. 1.75 to 1.8 Ga Changzhougou [41] and Chuanlinggou formations [42] of the lower part of the Changcheng Group, North China. The eukaryotic affinity of these palynomorphs is demonstrated by complex surficial micro-sculptural elements (fine striations), trilaminar ultrastructure of the vesicle wall, and longitudinal rupture of the vesicles, interpreted as an excystment feature. In our material, vesicle micro-sculptural elements, as well as wall ultrastructural morphology, might have been obliterated given the antiquity of these microfossils [43]. The FTIR and micro RAMAN spectra show similarities with those obtained for Mesoproterozoic organic-walled microfossils [44], although the Francevillian material is more aliphatic (Figures S13–S16 in File S2).

From the available dataset, the granular organic microstructures from the FB2 Subunit could represent the degraded remains of individual microorganisms. Given a complete absence of filaments, which would be indicative of benthic affinity, it seems reasonable to propose a planktonic origin for this assemblage.

## Discussion

The crucial issue with regard to the interpretation of the Francevillian specimens is not biogenicity as such, because microbial processes are almost always involved in sedimentary pyrite formation during early diagenesis. Instead, the question is whether the morphology observed in the specimens reflects the shape of fossilized macroscopic organisms or colonies, or whether it was formed by taphonomic/diagenetic processes. We have earlier argued [1], on grounds of crystallography and isotopic composition, that fossilization of the Francevillian organisms was a prolonged process, where the sheet fabric was original, preserved through microbially induced pyritization in an open environment, whereas the central lumps of pyrite, the cores, were formed in a close environment after burial as pyrite concretions. The new data confirm this model, but also add evidence for intermediate pyritization processes in some specimens (Fig. 11), where secondary growth of spongy pyrite modified the original structure but without forming a distinct core.

The biogenic morphology of the earlier reported sheet-like specimens is ostensibly challenged by their similarity with flat pyritic concretions such as pyrite “suns” and “flowers” [1]. Undulate or lobate outer edges, seen in such concretions as well as in the Francevillian sheets, can be created inorganically by fingering-driven Saffman-Taylor instability in a mixture of fluids with different viscosities [45]. However, the required pressure should then affect all comparable structures on the same bedding plane [45], which is not observed in the Francevillian material (Figure 3A, B). The textural and isotopic analysis of new specimens showing very different morphologies and the comparison with the two Phanerozoic pyrite concretions clearly demonstrate that the Francevillian specimens differ from the Phanerozoic pyrite concretions in both texture and isotopic composition and thus the latter cannot be used as a model for the formation of the former.

We have earlier proposed that the flexible sheets with radial fabric represent colonial organisms showing incipient multicellular organization [1]. The presence of lobate, elongated-to-rod-shaped, and discoidal structures, as well as the circular aggregation, expands the morphological diversity of the Francevillian organisms. The specimens showing a string connected to a sheet-shaped macrofossil (Figures 4C–F, 5, 6, Figure S1 in File S1) suggest that these two structures represent the same organism. A combination of elongated and flattened stages of life opens up the possibility that the organism had an organization similar to that of cellular slime mould, Dictyostelia. These organisms go through a “slug” phase in which amoeboid cells congregate into multicellular “slugs” that move along a mucus tube to a place where a sedentary fruiting body is formed [46], [3]. Dictyostelia are understood to branch from a deep position in eukaryote phylogeny [47]. The aggregational style of dictyostelid multicellularity, however, seems to confine them to the terrestrial environment [3], [48]. Given the strong evidence for a marine setting of the FB2 unit, Dictyostelia are therefore unsuitable even as an analogue of the behavior implied for the Francevillian organisms.

The occasional evidence for radial fabric in a narrow portion joined to a sheet-like morphology (Figures 6A–B; 4E–F) suggests that the rod-shaped portion does not represent a mucus tube, but an organic fabric similar to that which makes up the associated sheets. The difference with the rods lacking radial fabric may reflect preservation, but an alternative explanation is that the strings adjacent to a sheet in some cases represent a portion where the cells came to rest while still in the “slug” configuration.

The second most abundant member of the Francevillian biota is represented by the small non-pyritized to lightly pyritized disks (Figures 7A–F; 8A–B). With their positive relief and somewhat concentric and centrally radial texture, they are reminiscent of the small sand-volcano-like structures in the Cambrian King Square Formation in Canada [49]. Such an interpretation has the appeal of easily explaining why most of these disks are non-pyritized or weakly pyritized, while the other fossils from the Francevillian Series are pyritized. However, the Francevillian disks occur in organic matter-rich shales and are not directly associated with sandy or silty layers. Moreover, the Francevillian disks are generally smaller than the sand-volcano-like structures, and their profile is highly repetitive, while sand-volcano-like structures show variable morphologies [49]. The Francevillian disks are therefore likely not related to fluid escape. The size and morphology of the Francevillian disks also resemble some Ediacaran disks [50], however, they differ from the latter as they appear in positive epirelief, while most Ediacaran disks are preserved in positive hyporelief. Moreover, the morphology of the Francevillian disks does not exactly correspond to any of the described Ediacaran disks [50]. Finally, the Francevillian disks are somewhat similar to the giant Proterozoic acritarch *Chuaria* [51], but differ in their larger size and radially striated centre. Partial pyritization of some of the Francevillian disks gives support to their original organic composition, but the scarcity of this pyritization also suggests that their nature was somewhat different from that of the pyritized specimens.

In its organization, the circular aggregate shown in Figures 4F–G and 5F–G is reminiscent of the tight assemblage of circular structures of *Nemiana* and *Beltanelloides* [52]. *Nemiana*, however, appears in high positive hyporelief, while the Francevillian aggregate is preserved in low positive epirelief. Moreover, although *Nemiana* and *Beltanelloides* subunits tend to be packed in a similar way, they do not form discrete rounded aggregates. A superficial resemblance also exists to the trace fossil *Paleodictyon nodosum*, an enigmatic structure observed at the surface of deep-sea sediments [53], [54]. Though *Paleodictyon* is mostly described from Tertiary flysch sequences, it has a larger stratigraphic distribution, and its oldest occurrences are in Cambrian sediments [54]–[56]. *Paleodictyon* generally corresponds to a strictly hexagonal network of tunnels or tubes delineating isodiametric hexagons seen in positive hyporelief. Compared to *Paleodictyon nodosum*, the Francevillian fossil consists of rounded bodies of different sizes, the packing of which only occasionally produces a hexagonal pattern.

Overall, the Francevillian biota represents an exceptional Paleoproterozoic oxygenated ecosystem [1], [18] comprising several types of macroscopic organisms, including the pyritized fossils, non-pyritized or lightly pyritized disks and circular aggregates, as well as carbonaceous microorganisms. As illustrated by the sulfur isotope record, early pyritization in a quiet depositional setting [21] is likely the most critical factor responsible for the exceptional preservation of these fossils.

The emergence of this biota follows a rise in atmospheric oxygen, which is consistent with the idea that surface oxygenation allowed the evolution and ecological expansion of complex megascopic life [57], [58], [39], [18]. The disappearance of the Francevillian macrofossils in the upper part of the FB2b black shales is apparently related to increased energy in the environment. The macrofossils are not observed in the overlying Francevillian black shales of the FD Formation that were deposited under an anoxic and sulfidic (euxinic) water column [18]. Their absence from the later fossil record might ultimately be related to the fall in the atmospheric oxygen level that followed the ca. 2.22-2.1 Ga Lomagundi carbon isotope excursion [18], [59]–[63], followed by a long-lived and extensive marine anoxia that forms the hallmark for the most of the Proterozoic Era after ca. 2.1 Ga [59], [18]. Oxygen content in surface environments is not universally accepted as a major driver for the evolution and complexification of multicellular life [64], [65]. However, the emergence and later disappearance of megascopic life in association with oxygen overshoot and fall in the early Paleoproterozoic Eon [18], [59]–[63] is consistent with oxygen availability as a driver of evolutionary adaptation [39], [66], including aspects of body size [67].

The Francevillian deposits represent exceptionally well-preserved Paleoproterozoic sedimentary rocks deposited in shallow-marine, oxygenated environment [11], [12], [17]. This, coupled with pyritization during early diagenesis, provides a unique window on the early Paleoproterozoic biosphere during one of the most critical time periods in Earth's history. The Francevillian biota formed a diverse ecosystem. It appears to represent a first experiment in megascopic multicellularity.

## Supporting Information

Table S1 **Sulfur isotopes data (δ^34^S).**
(XLSX)

File S1 **Sulfur isotopes: Data of sulfur isotopes performed on nine specimens variable in morphostructure and dimensions.** For comparison, we also analyzed the data of two types of pyrite concretions in Phanerozoic rocks.
(PDF)

File S2 **Spectroscopic analyses of Acritarchs: Data obtained by XANES, FTIR and RAMAN; in order to characterize the organic matter.**
(PDF)
