# Supplementary material for: The 2.1 Ga Old Francevillian Biota: Biogenicity, Taphonomy and Biodiversity
Source: PLoS One. 2014 Jun 25;9(6):e99438. doi: 10.1371/journal.pone.0099438 (PMC4070892; doi:10.1371/journal.pone.0099438)
Supplement: File S1 — Sulfur isotopes: Data of sulfur isotopes performed on nine specimens variable in morphostructure and dimensions. For comparison, we also analyzed the data of two types of pyrite concretions in Phanerozoic rocks. (PDF) [file pone.0099438.s002.pdf]

Supporting Information

Sulfur isotope ($\delta^{34}S$) analyses

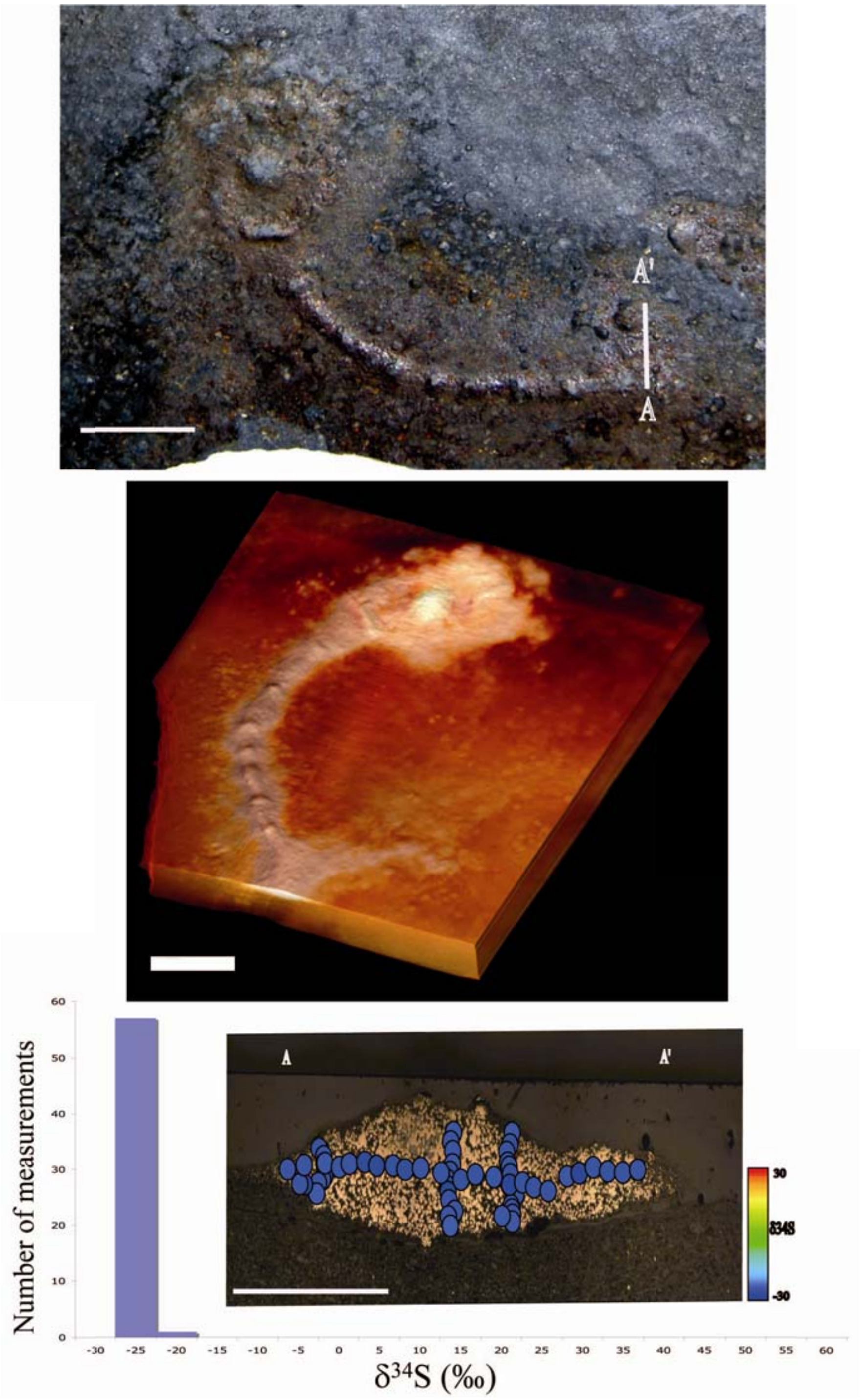

Figure S1. The A-A' section through the specimen G-FB2-s-259 and the corresponding histogram of $\delta^{34}S$ values (cf. Table S1). Distribution of S isotope values in the map was rendered following a color scale increasing from dark blue to red. Scale bars are 1.0 cm.

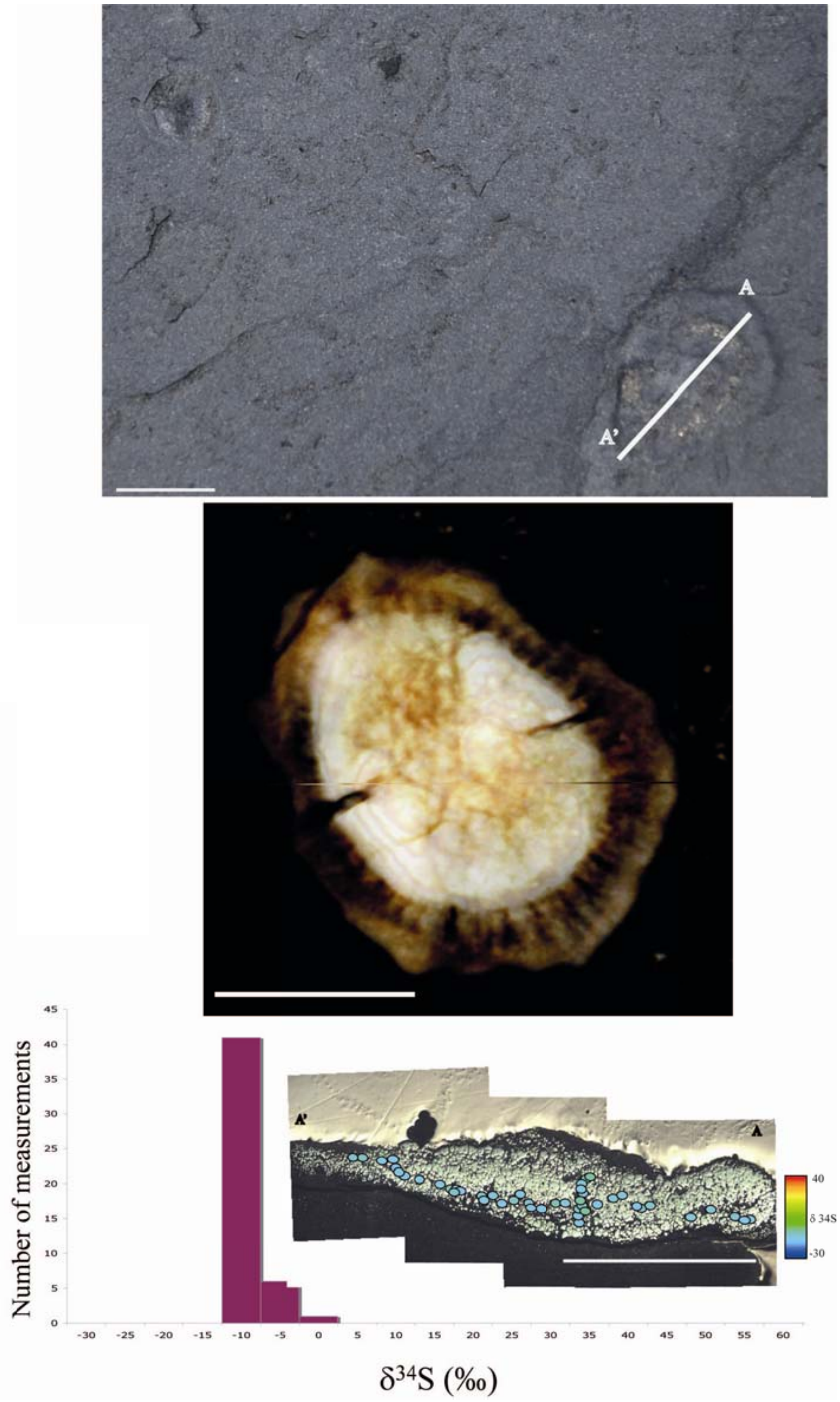

Figure S2. The A-A' section through the specimen G-FB2-s-223 (the circular lines in the CT picture are artefacts of the tomographic rendering) and the corresponding histogram of $\delta^{34}S$ values (cf. Table S1). Distribution of S isotope values in the map was rendered following a color scale increasing from dark blue to red. Scale bars are 1.0 cm.

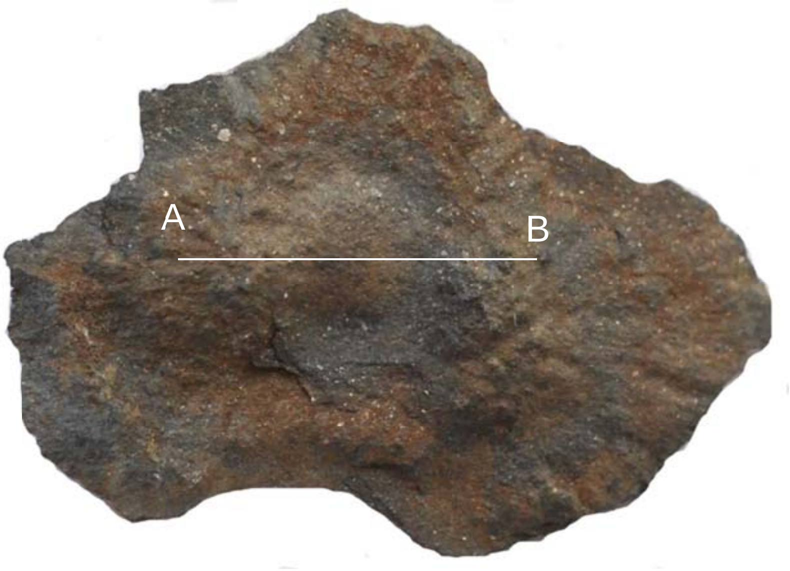

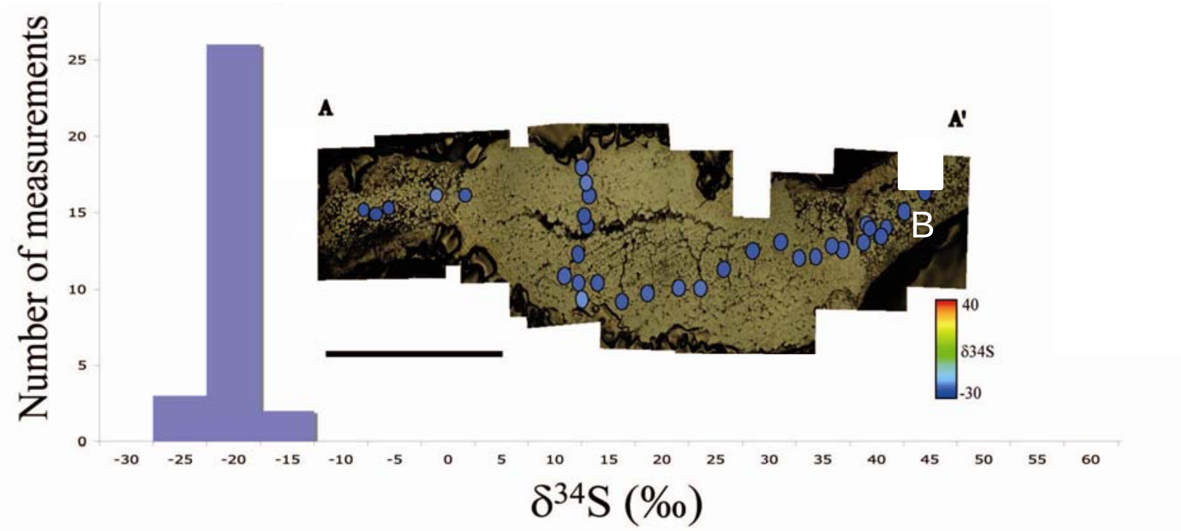

Figure S3. The A-A' section through the specimen G-FB2-s-272 and the corresponding histogram of $\delta^{34}S$ values (cf. Table S1). Distribution of S isotope values in the map was rendered following a color scale increasing from dark blue to red. Scale bars are 1.0 cm.

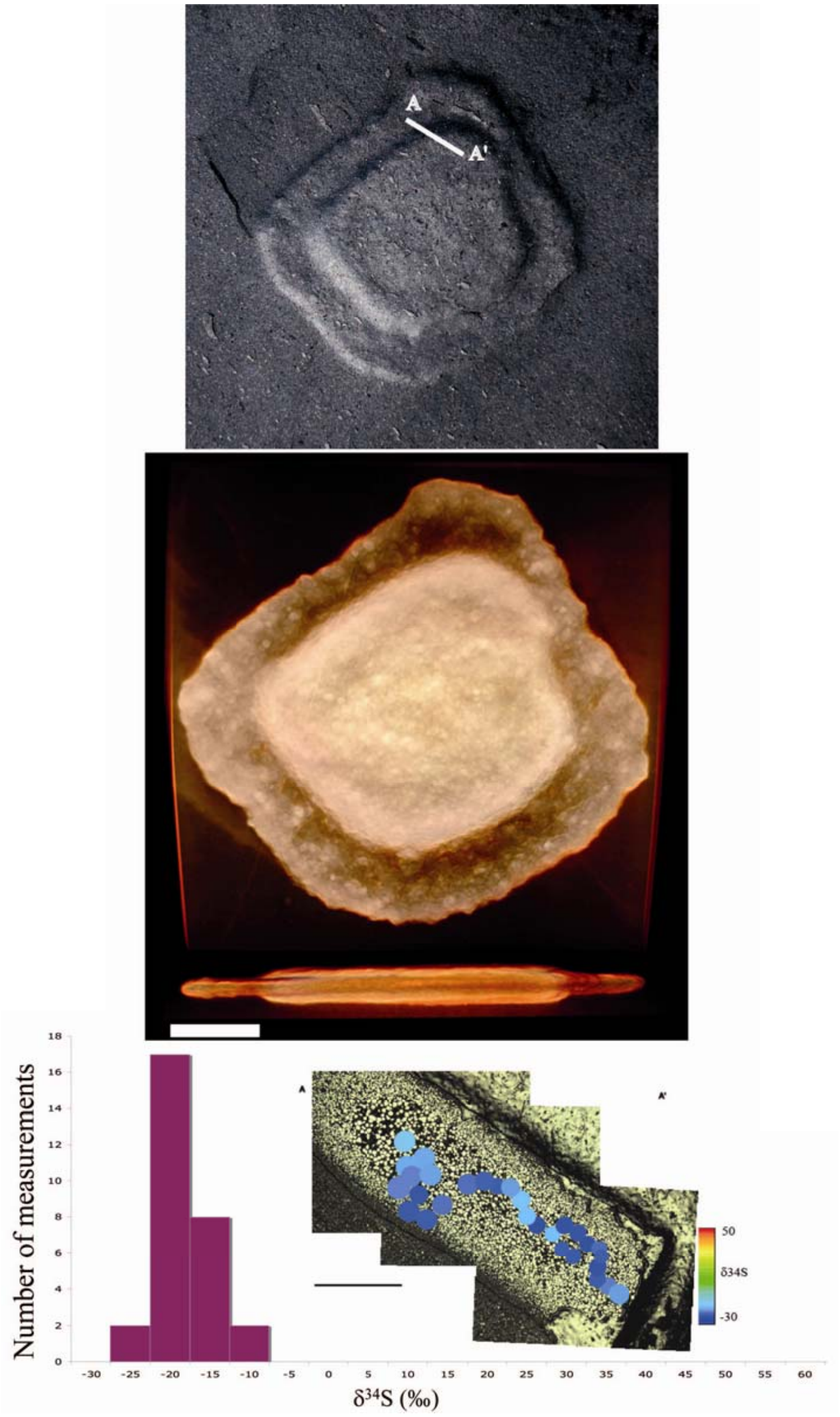

Figure S4. The A-A' section through the specimen G-FB2-s-573 and the corresponding histogram of $\delta^{34}S$ values (cf. Table S1). Distribution of S isotope values in the map was rendered following a color scale increasing from dark blue to red. Scale bars are 1.0 cm.

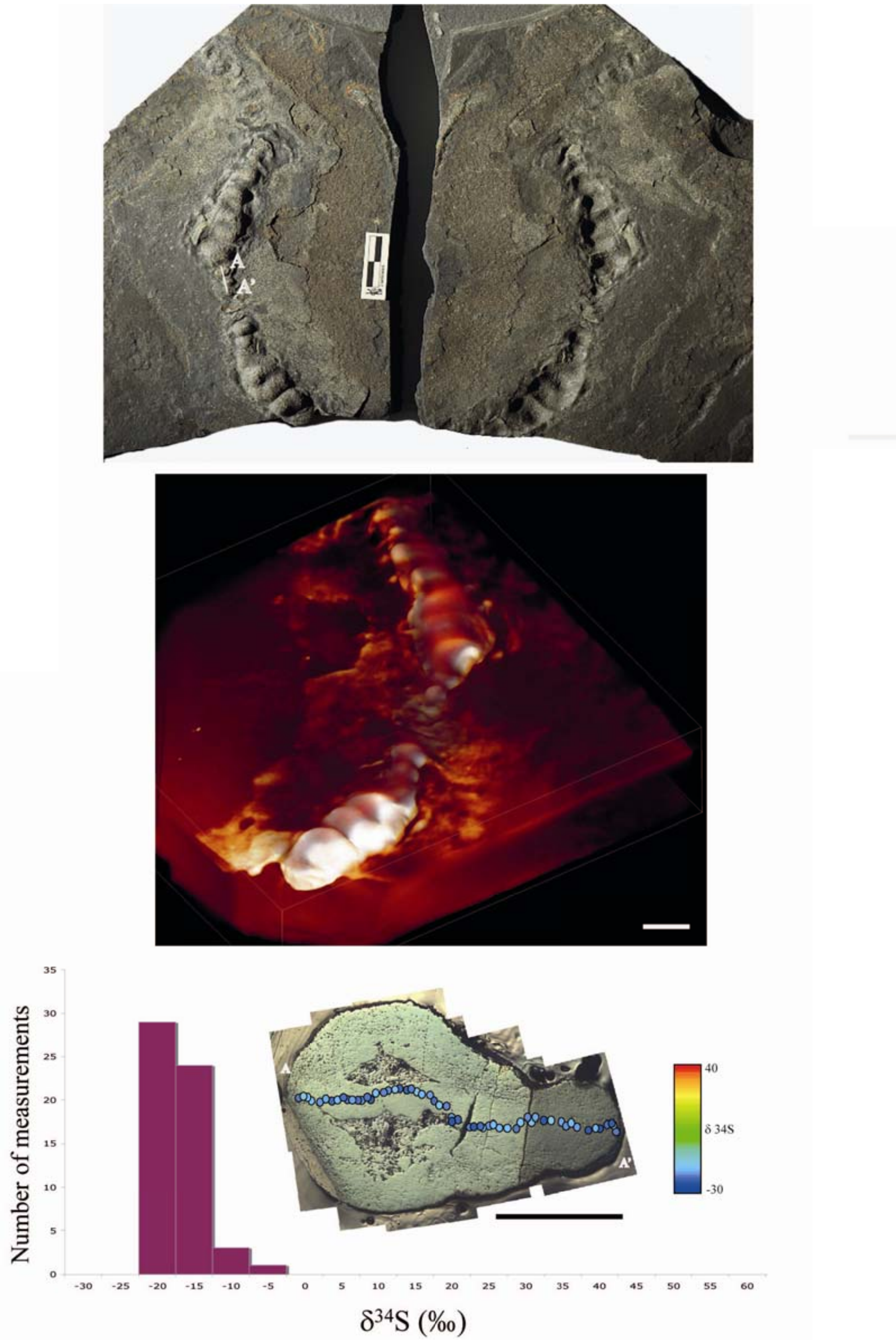

Figure S5. The A-A' section through the specimen G-FB2-s-71 and the corresponding histogram of $\delta^{34}S$ values (cf. Table S1). Distribution of S isotope values in the map was rendered following a color scale increasing from dark blue to red. Scale bars are 1.0 cm.

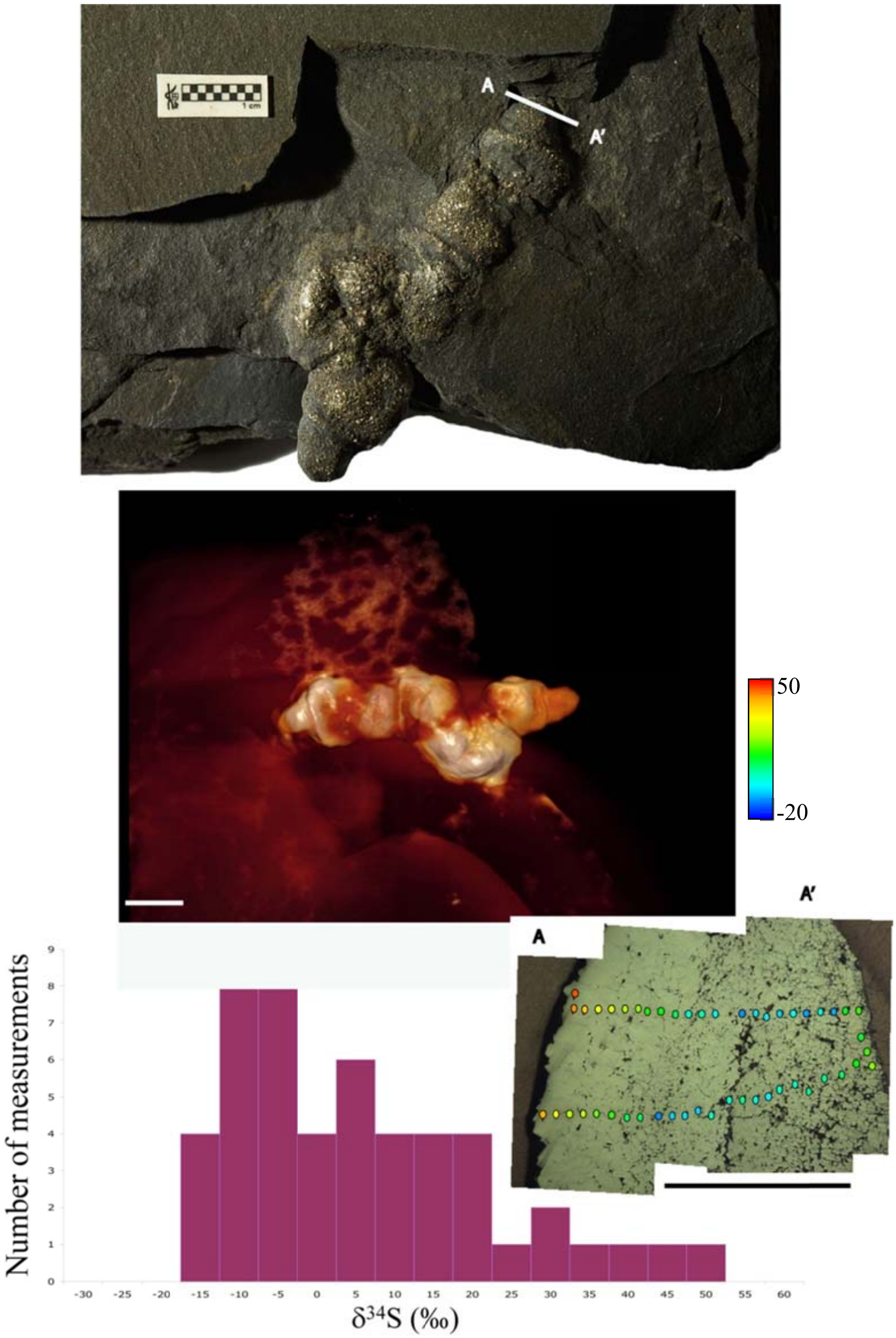

Figure S6. The A-A' section through the specimen G-FB2-s-576 and the corresponding histogram of $\delta^{34}S$ values (cf. Table S1). Distribution of S isotope values in the map was rendered following a color scale increasing from dark blue to red. Scale bars are 1.0 cm.

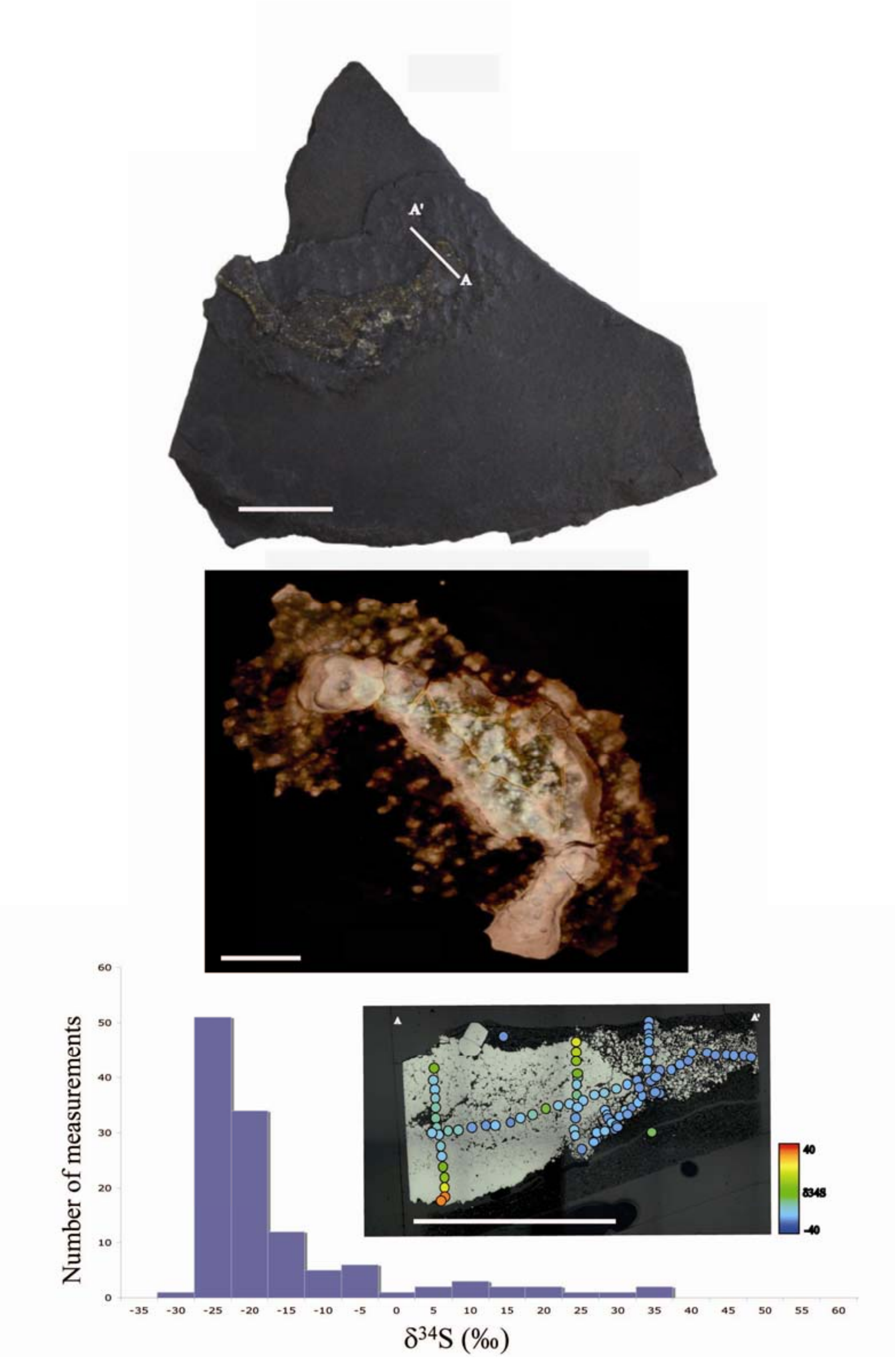

Figure S7. The A-A' section through the specimen G-FB2-s-52 and the corresponding histogram of $\delta^{34}S$ values (cf. Table S1). Distribution of S isotope values in the map was rendered following a color scale increasing from dark blue to red. Scale bars are 1.0 cm.

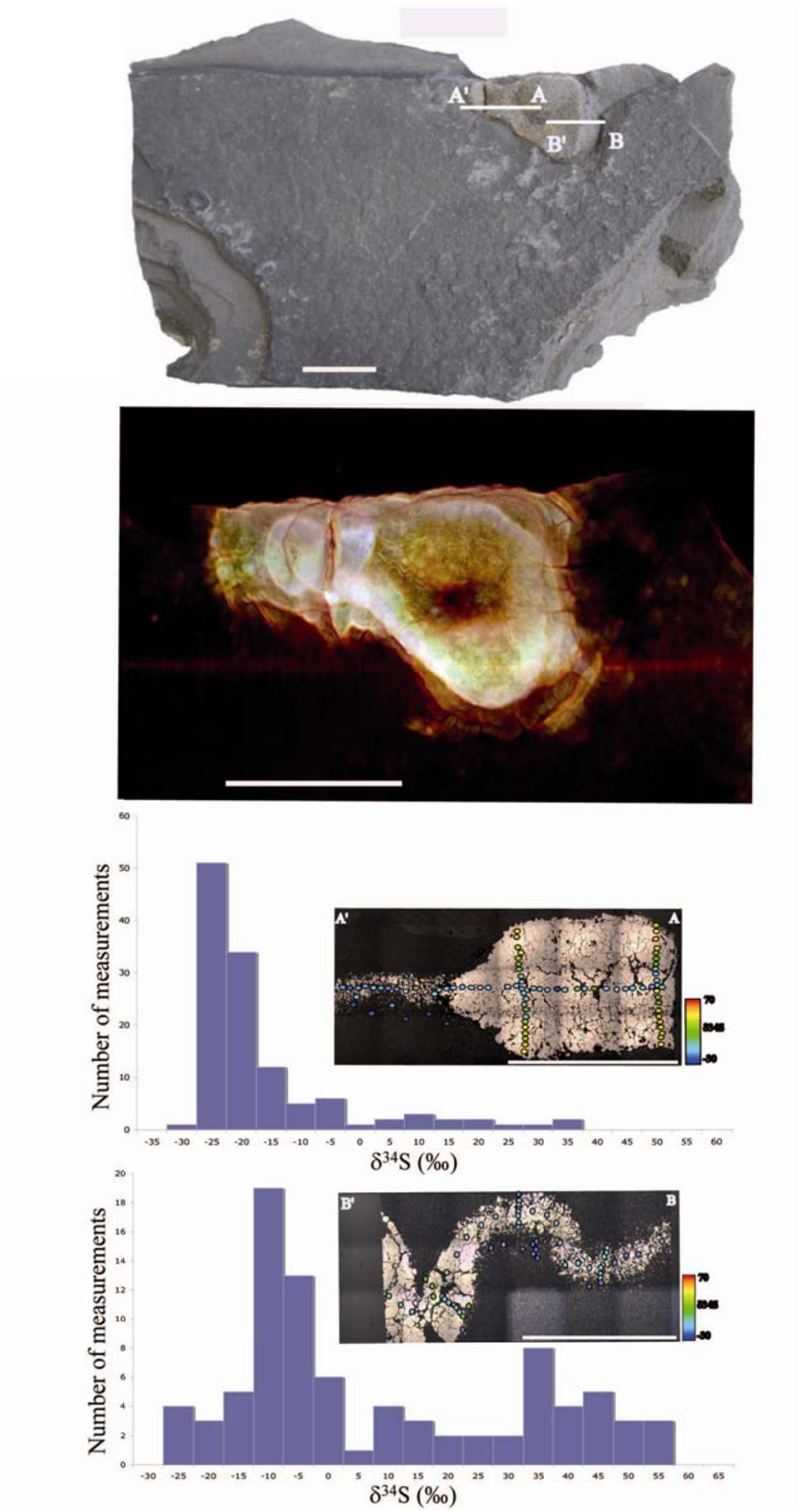

Figure S8. The A-A' and B-B' sections through the specimen G-FB2-s-57 and the corresponding histograms of $\delta^{34}S$ values (cf. Table S1). Distribution of S isotope values in the maps was rendered following a color scale increasing from dark blue to red. Scale bars are 1.0 cm.

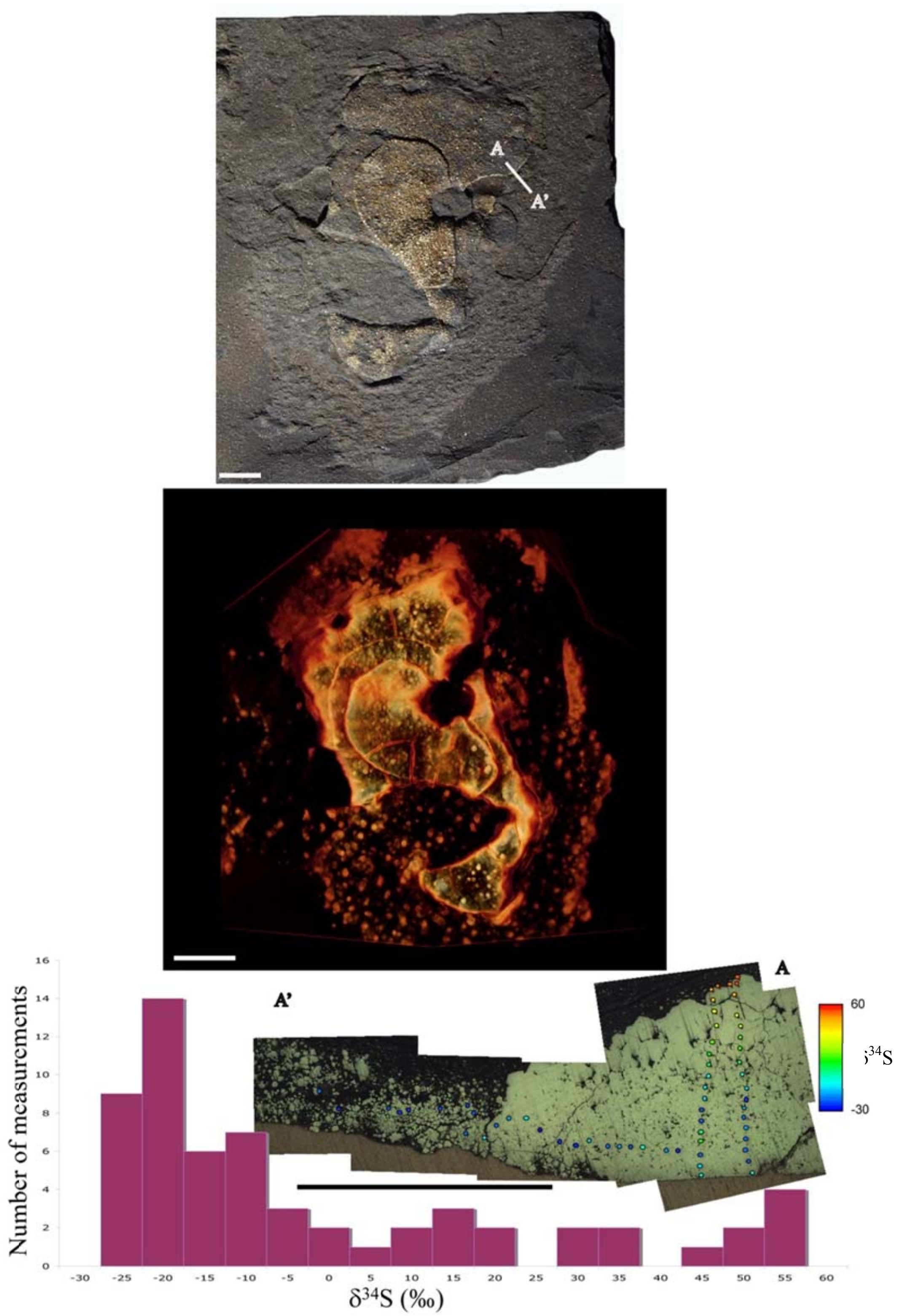

Figure S9. The A-A' section through the specimen G-FB2-s-575 and the corresponding histogram of $\delta^{34}S$ values (cf. Table S1). Distribution of S isotope values in the map was rendered following a color scale increasing from dark blue to red. Scale bars are 1.0 cm.

1.2 - Analysis of a Carboniferous pyrite sun and of a Jurassic pyrite flower

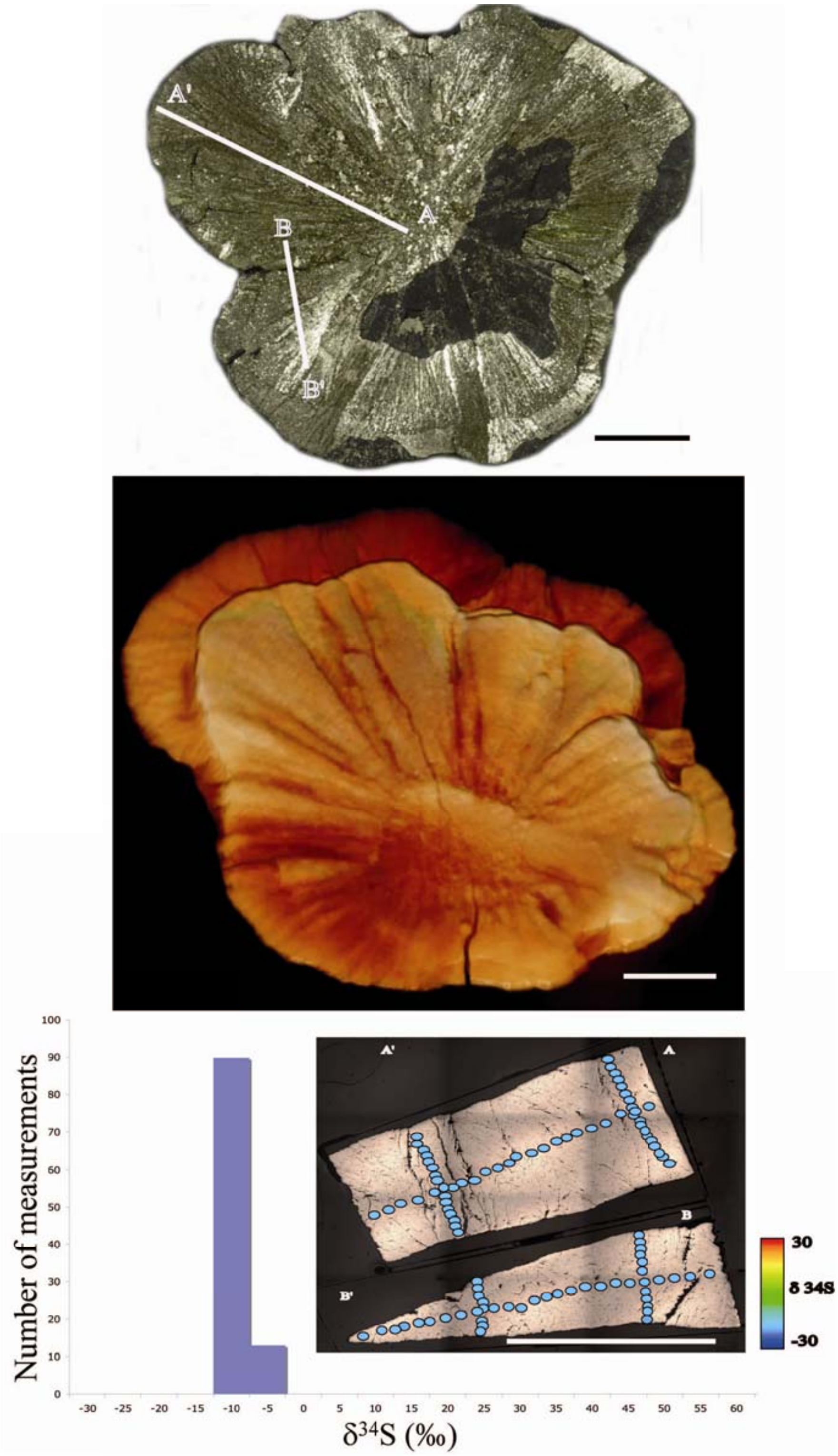

Figure S10. The A-A' and B-B' sections through the Upper Carboniferous pyrite sun of Sparta, Illinois (Swedish museum number SMNH X4450) and the corresponding histograms of $\delta^{34}S$ values (cf. Table S1). Distribution of S isotope values in the maps was rendered following a color scale increasing from dark blue to red. Scale bars are 1.0 cm.

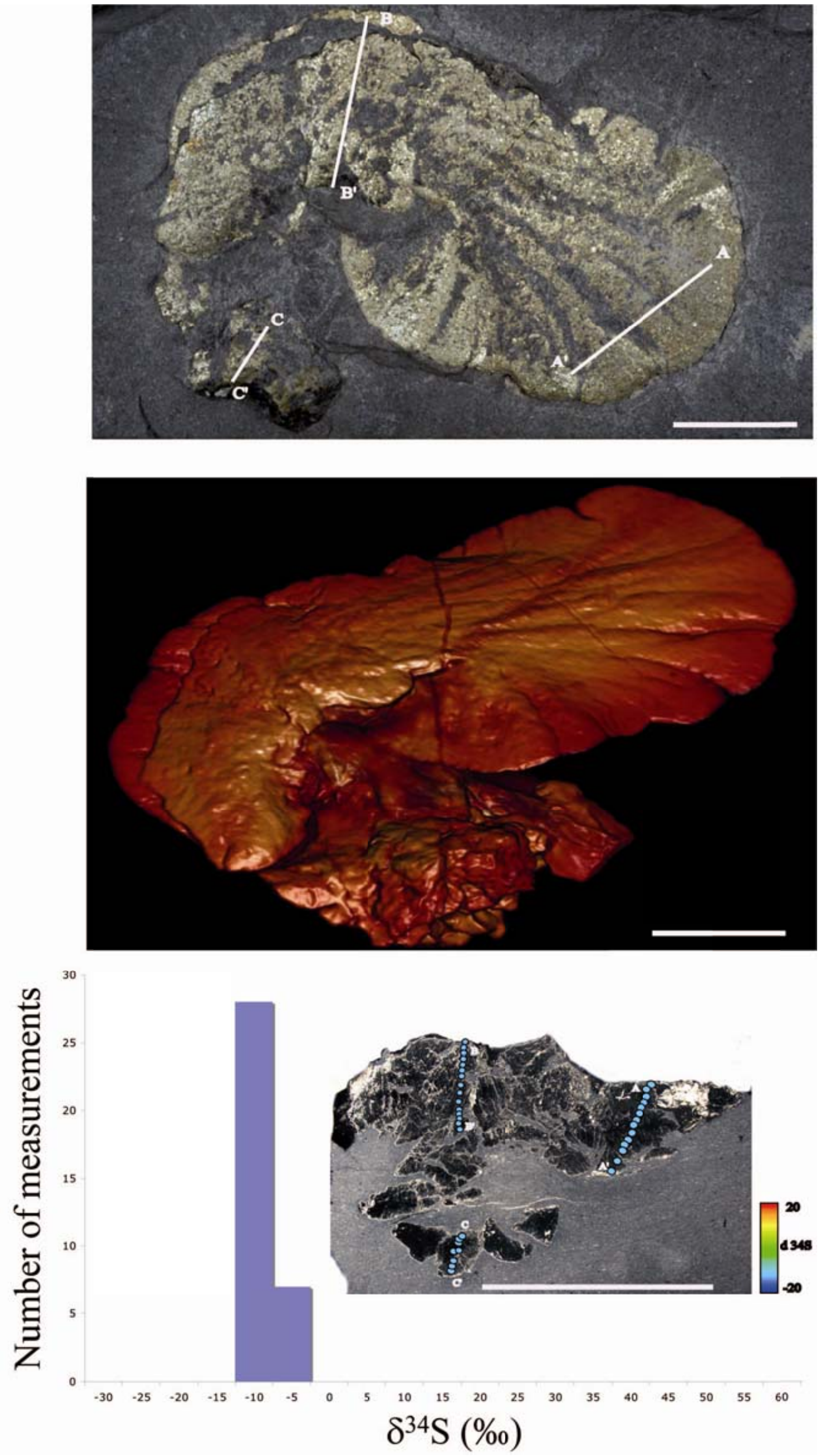

Figure S11. The A-A', B-B' and C-C' sections through the pyrite flower from the Upper Jurassic Posidonia Shale, southern Germany (Swedish museum number SMNH X4449), and the corresponding histograms of $\delta^{34}S$ values (cf. Table S1). Distribution of S isotope values in the maps was rendered following a color scale increasing from dark blue to red. Scale bars are 1.0 cm.
