# Supplementary material for: The 2.1 Ga Old Francevillian Biota: Biogenicity, Taphonomy and Biodiversity
Source: PLoS One. 2014 Jun 25;9(6):e99438. doi: 10.1371/journal.pone.0099438 (PMC4070892; doi:10.1371/journal.pone.0099438)
Supplement: File S2 — Spectroscopic analyses of Acritarchs: Data obtained by XANES, FTIR and RAMAN; in order to characterize the organic matter. (PDF) [file pone.0099438.s003.pdf]

Spectroscopic analyses of acritarchs

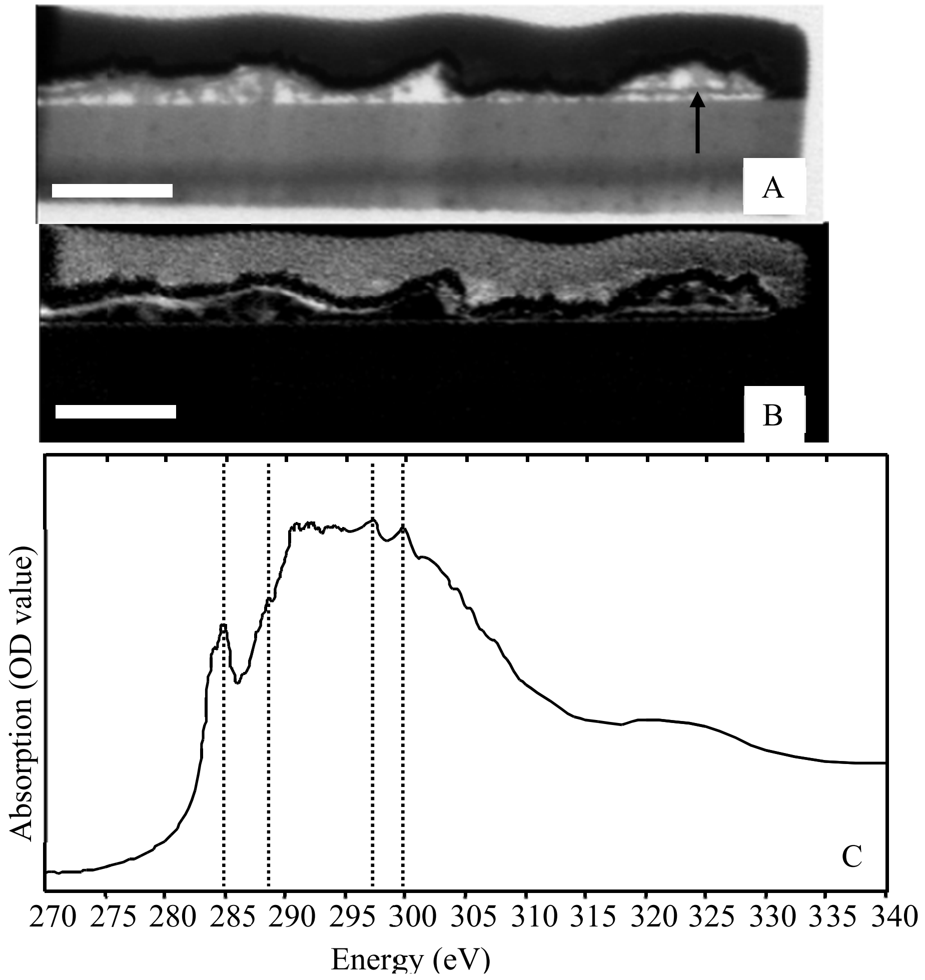

Figure S12. Scanning transmission X-ray microscopy analysis of the FIB foil at the C K-edge. (*A*) Image of the FIB foil above the C K-edge at 288.6 eV. The platinum and gold layers appear in dark. The glass coverslip appears in grey at the bottom. The acritarch can be seen as a thin line (black arrow). (*B*) Map of organic carbon obtained by subtracting the image at 280 eV converted from the image at 285 eV. Organic carbon is present in the platinum strap. The acritarch can be seen as a bright organic carbon-rich film beneath the platinum and gold layers. (*C*) X-ray absorption near edge structure (XANES) spectrum at the C K-edge obtained on the acritarch. Vertical dotted lines at 284.9, 288.6, 297.2 and 299.9 eV are attributed to electronic transitions 1s→π* in aromatic functional groups, 1s→ π* in carboxylic functional groups and L3 and L2 edges of potassium present in the minerals, respectively. Absorption by aliphatic carbon may be masked by the absorption by other functional groups. Scale bar is 2 µm.

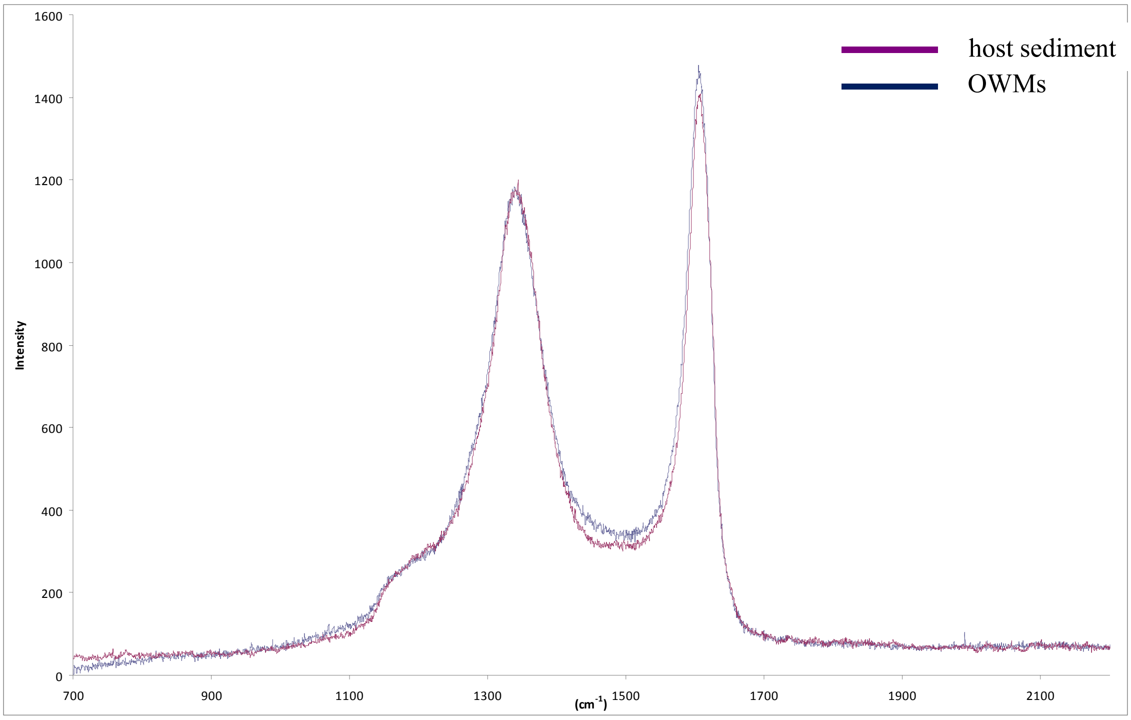

Figure S13. First-order Raman spectrum of carbonaceous spheroidal microstructures (OWMs) measuring 50-80 μm in diameter and their host sediment. Band assignments: D band - 1.355 cm^{-1}; G band - 1.590 cm^{-1}.

<u>FTIR spectra of acritarchs</u>

Assignment of absorption bands (Figs S14-S16)

The full spectra of the specimens are presented at the bottom of Figs S14 and S16.

- The large band centred at 3150-3100 cm^{-1} corresponds to OH groups from organic matter (alcohol or acid functions) and/or adsorbed water.
- The doublet between 3000 and 2800 cm^{-1} corresponds to aliphatic absorptions by CH, CH_2 and CH_3 stretching. The most important bands are related to CH_2, indicating the presence of aliphatic chains. The shoulders, related to CH_3 are relatively important.
- The carbon double bonds absorb between 1800 and 1500 cm^{-1}. The band at 1710 cm^{-1} corresponds to C=O stretch of carboxylic acid groups. The bands at 1660 and 1630 cm^{-1} are related to C=O bonds conjugated to other double bonds (e.g., quinones). The absorption at 1590 cm^{-1} is related to aromatic C=C bonds.
- The bands between 1480 and 1300 cm^{-1} are mostly related to CH_2 and CH_3 deformation.
- Absorptions in the 1090-1200 cm^{-1} region can be attributed to C-O stretching (ethers, alcohols), in particular from carbohydrates/polysaccharides.

Deconvolution of C-H_x stretching bands (Fig. S15) (28) suggests a CH_2/CH_3 ratio of 2.46 for specimen 1 and 2.50 for specimen 2. These values indicate a certain degree of branching and/or relatively short chain aliphatics (1). The proxy has been poorly tested, however, and the influence of CH from aromatics and CH_2 of rings is incompletely known. The absence of a band at 720 cm^{-1} confirms the absence of long aliphatic chains.

Comparison with other spectra

The acritarch wall macromolecule contains relatively short carbon chains (on average <C8 according to (1), a significant proportion of oxygen in form of C-O-C and C=O (quinones), and aromatic rings.

The organic matter in the Gabonese sediments is relatively mature (maximum reflectance values often >4%) (2, 3). Upon maturation, removal of oxygen and hydrogen takes place so that the material may aromatize and characteristic features of the FTIR spectra disappear (4). Two relevant points arise from the obtained spectra: (a) how do they compare to other palynomorphs of similar age, and (b) what was the original wall biomolecule and how does it compare with the biopolymers we know today.

Comparison with FTIR spectra from Proterozoic palynomorphs

Three of the four palynomorphs from the Neoproterozoic of Australia are relatively aliphatic (5, 6), while *Leiospheridia* sp. is more aromatic. The 3 acritarchs from the Mesoproterozoic of China and of Australia appear relatively aromatic (5), a feature which the authors mainly relate to the original composition of the material and not to thermal maturity. The organic Meso- to Neo-Proterozoic macrofossil *Chuaria* appears to contain both longer aliphatic chains, but also more distinct aromatic rings (7).

Origin of the material. Comparison to present-day polymers

Although the possibility exists that the acritarchs considered here have been produced by a unique, presently extinct biosynthetic pathway, we do not assume this *a priori*. Recent organisms synthesize only a relatively small number of degradation-resistant wall polymers, such as cellulose, chitin, algaenan and sporopollenin. These polymers have a wide phylogenetic distribution, suggesting an ancient origin. It is therefore likely that these pathways also gave rise to the Gabonese acritarchs. Possible exceptions are the polymers unique to organisms living on land, which may have evolved during the Phanerozoic. Three groups of resistant biopolymers can be distinguished.

The first group uses carbohydrates as the most important constituents of the cell-wall biopolymers. Cellulose is a pure carbohydrate polymer and its IR spectrum is dominated by O-H (3100-3600 cm^{-1}) and C-O (900-1200 cm^{-1}) absorption bands. Cellulose is widespread in nature, especially in cell walls of higher plants and algae (8). A material similar to cellulose composes the cysts of some dinoflagellates (9). Although some bacteria secrete cellulose, cellulose walls are a eukaryotic feature. Chitin is also made by Eukaryotes. It is widespread in skeletons and shells of arthropods, but is also present in fungi. Although some algae, e.g., the green alga Chlorella (10), two diatom species (11, 12), and the haptophyte Phaeocystis (13), also produce chitin, chitin production appears mainly associated to a heterotrophic lifestyle. Chitin has 2-amino glucose as its basic element. Its FTIR spectrum has strong absorptions associated with amide N-H stretch (3100 cm^{-1}), amide I C=O stretch (1630 cm^{-1}), and C-N stretch in the amide II (1550 cm^{-1}) and amide III (1300 cm^{-1}). The prokaryotic Bacteria synthesize cell and spore-walls made of peptidoglycans, polymers made of glycan strands crosslinked by peptides. Peptidoglycan FTIR spectrum presents similarities with chitin spectrum with strong absorptions related to sugar and amide functions (14). For Archaea, four different wall types are known (15). The walls may consist of polysaccharides only, mixtures of sugars and amino acids (pseudopeptidoglycan, glycoprotein), or proteins only.

A second set of wall biopolymers is based on cross-linked long aliphatic carbon chains. Algaenan is the best example. It widespread in the cell walls of green algae and has been reported from some Eustigmatophytes and a motile dinoflagellate (16). In algaenan, the long aliphatic chains are linked by ether or ester bonds. Its FTIR spectrum is largely dominated by absorptions of long aliphatic chains. Cutin and cutan present in higher plants are also of this type (16).

A third group of wall polymers includes phenylpropanoids (8, 16). Among these is sporopollenin, which composes the cell walls of higher plant spores and pollen. It is based on ether-linked propyl-phenol moieties and fatty acids. Its spectrum shows a contribution from aliphatic groups, but also typical absorptions related to the isolated benzene rings at 1510 and 820 cm^{-1}. Lignin, produced by vascular plants, is basically a propyl-methoxyphenol polymer. To date, it seems that the production of aromatic biopolymers is associated to a terrestrial life-style and may relate to the need for UV protection, skeletal resistance to gravity or resistance to desiccation in aerial environments.

Comparison of acritarch spectra with these biopolymers (Fig. S16) shows that the acritarchs correspond to neither of these materials. This is consistent with the long diagenetic and thermal history of the organic matter in the Francevillian sediments. During early diagenesis, aliphatic chains may get attached to the wall macromolecules through oxidative polymerisation and/or sulfurisation. This may remain largely undetected in the case of aliphatic polymers like algaenan; however, this process is clearly observed in the carbohydrate containing aromatic biopolymers (16). Upon thermal maturation, in the oil window, the long aliphatic chains originally present or added during early diagenesis become released. The original long-chain biopolymers disintegrate into oil, while aromatization is mostly associated to carbohydrate-based (e.g., cellulose and chitin), and aromatic (e.g., sporopollenin) biopolymers.

Considering that aromatic biopolymers most probably evolved in the Phanerozoic, sporopollenin can be safely excluded as original material for the Gabonese acritarchs. The acritarch spectra here observed are therefore interpreted as reflecting a carbohydrate-based polymer, which upon diagenesis included variable proportion of aliphatic material such as fatty acids. However, our understanding of diagenetic processes does not allow to assess if nitrogen-containing groups were originally present. The FTIR spectra could also reflect an algaenan-type material that has suffered partial aromatisation due to thermal maturation. Though this second possibility appears less likely, it cannot be fully excluded.

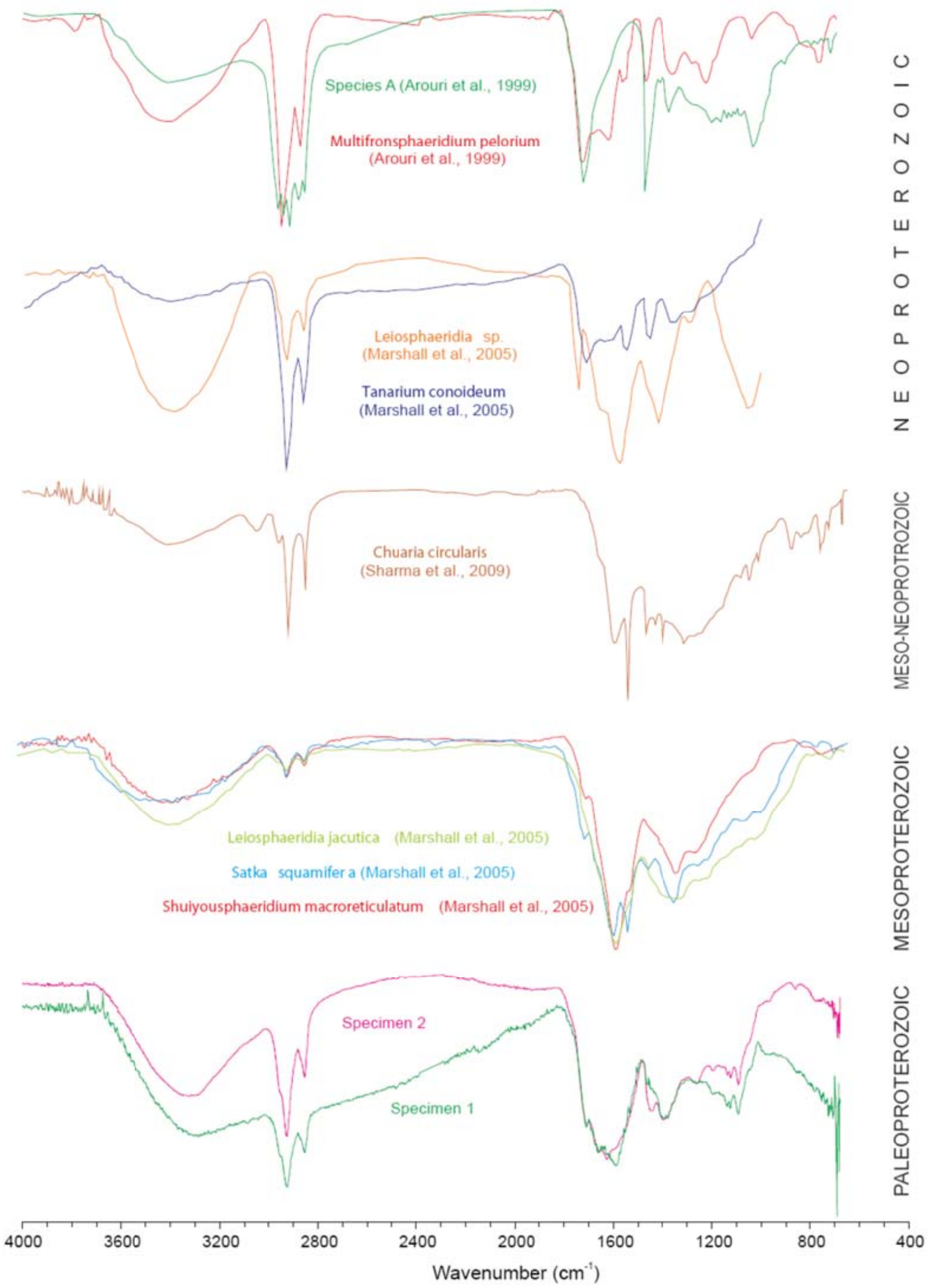

Figure S14. FTIR spectra of two acritarchs (spec. 1 and 2 [bottom]) from the FB2 of Gabon compared to the spectra of different Proterozoic organic microfossils; compiled data from (4), (6) and (7).

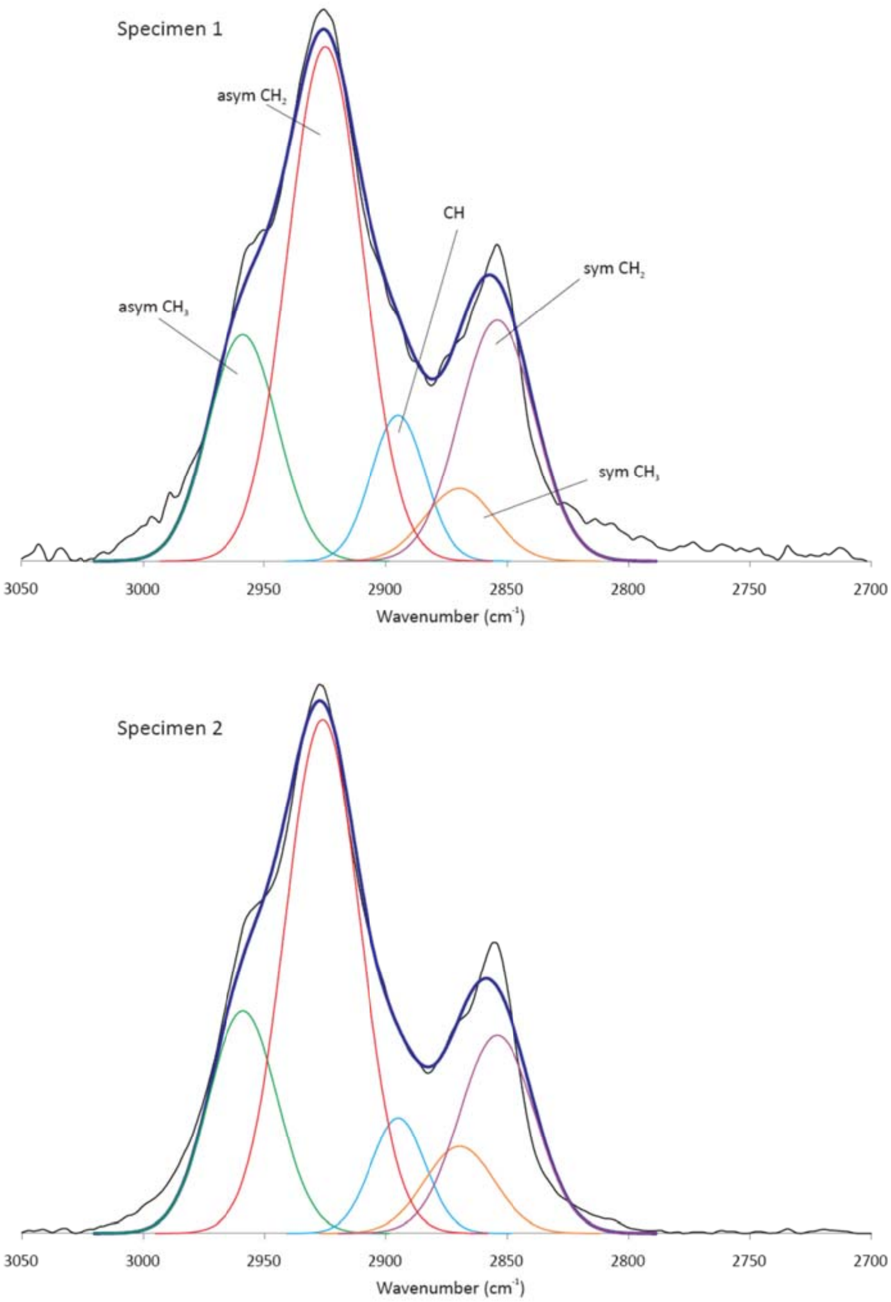

Figure S15. Deconvolution of C-H_x stretching bands (3000-2800 cm^{-1}) of the individual FTIR spectra of two acritarchs (spec. 1 [upper] and spec. 2 [lower]) from the FB2 of Gabon.

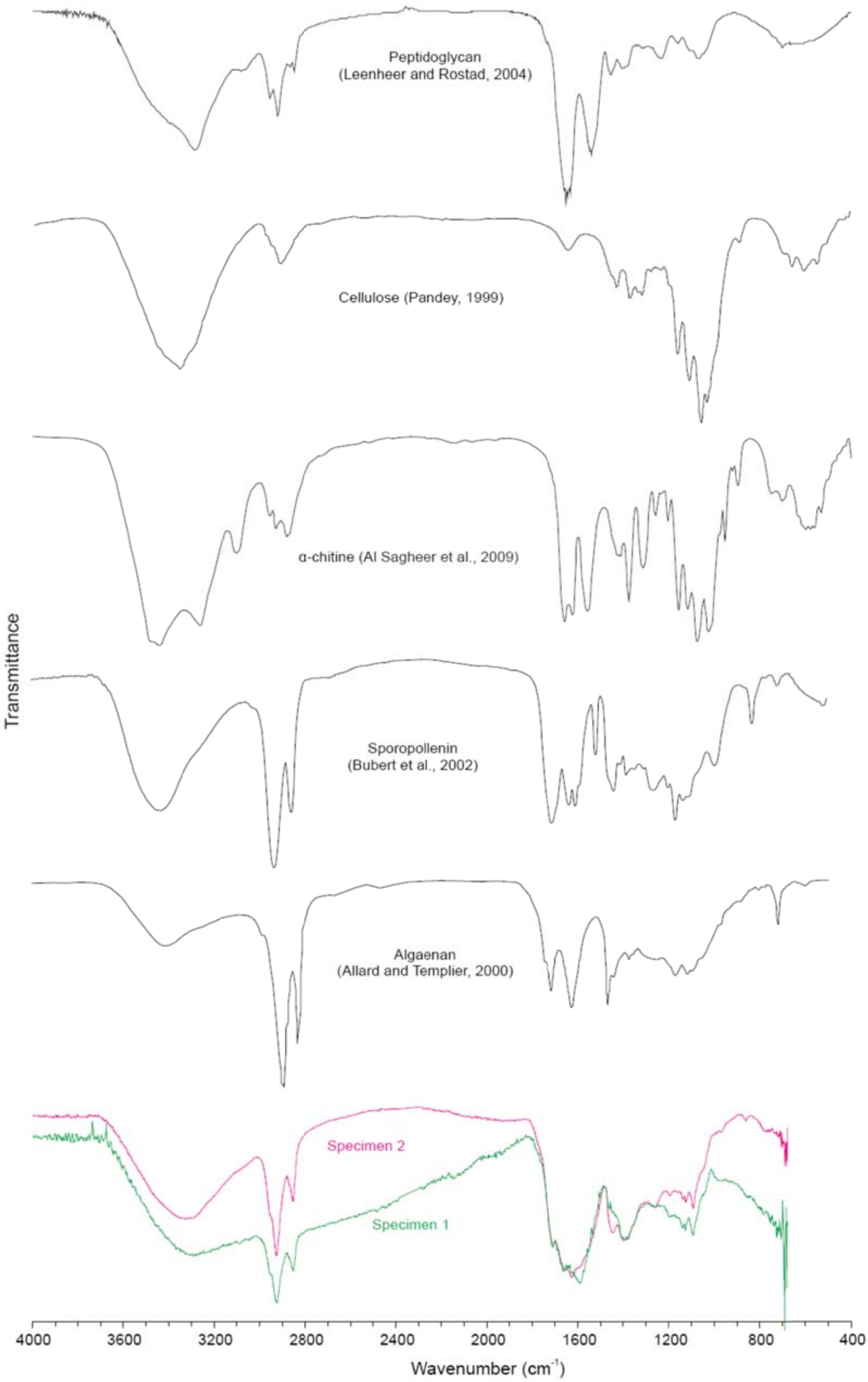

Figure S16. FTIR spectra of two acritarchs (spec. 1 and 2 [bottom]) from the FB2 of Gabon compared to the spectra of common present-day biopolymers; compiled data from (17), (18), (19), and (20).

References

1. Lin R, Ritz GP (1993) Studying individual macerals using i.r. microspectrometry, and implications on oil versus gas/condensate proneness and “low-rank” generation. *Org Geochem* 20:695–706

2. Cortial F, Gauthier-Lafaye F, Lacrampe-Couloume G, Oberlin A, Weber F (1990) Characterization of organic matter associated with uranium deposits in the Francevillian formation of Gabon (Lower Proterozoic). *Org Geochem* 15:73–85.

3. Mossman DJ, Nagy B, Rigali MJ, Gauthier-Lafaye F, Holliger P (1993) Petrography and paragenesis of organic matter associated with the natural fission reactors at Oklo, Republic of Gabon: a preliminary report. *Int J Coal Geol* 24:179–194.

4. Marshall CP, Javaux EJ, Knoll AH, Walter MR (2005) Combined micro-Fourier transform infrared (FTIR) spectroscopy and micro-Raman spectroscopy of Proterozoic acritarchs: A new approach to Palaeobiology. *Precambrian Res* 138:208–224.

5. Tissot BP, Welte DH (1984) *Petroleum formation and occurrence* (Springer-Verlag, Berlin).

6 Arouri K, Greenwood PF, Walter MR (1999) A possible chlorophycean affinity of some Neoproterozoic acritarchs. *Org Geochem* 30:1323–1337.

7. Sharma M, Mishra S, Dutta S, Banerjee S, Shukla Y (2009) On the affinity of *Chuaria–Tawuia* complex: A multidisciplinary study. *Precambrian Res* 173:123–136.

8. De Leeuw JW, Largeau C (1993) in *Organic Geochemistry*, eds Engel MH, Macko SA (Plenum Press, New York).

9. Versteegh GJM et al. (2012) Infra red spectroscopy, flash pyrolysis, thermally assisted hydrolysis and methylation (THM) in the presence of tetramethylammonium hydroxide (TMAH) of cultured and sediment-derived *Lingulodinium polyedrum* (Dinoflagellata) cyst walls. *Org Geochem* 43:92–102.

10 Kapaun E, Reisser W (1995) A chitin-like glycan in the cell wall of a *Chlorella* sp. (Chlorococcales, Chlorophyceae). *Planta* 197:577–582.

11 Herth W, Zugenmaier P (1977) Ultrastructure of the chitin fibrils of the centric diatom *Cyclotella cryptica*. *J Ultrastruct Res* 61:230–239.

12 Durkin CA, Mock T, Armbrust EV (2009) Chitin in diatoms and its association with the cell wall. *Eukaryot Cell* 8:1038–1050.

13 Ogawa Y, Kimura S, Wada M, Kuga S (2010) Crystal analysis and high-resolution imaging of microfibrillar α-chitin from *Phaeocystis*. *J Struct Biol* 171:111–116.

14 Naumann D, Barnickel G, Bradackzek H, Labischinski H, Giesbrecht P (1982) Infrared Spectroscopy, a Tool for Probing Bacterial Peptidoglycan. *Eur J Biochem* 125:505–515.

15 Kandler O, König H (1998) Cell wall polymers in Archaea (Archaebacteria). *Cell Mol Life Sci Cmls* 54:305–308.

16 De Leeuw JW, Versteegh GJM, van Bergen PF (2006) Biomacromolecules of algae and plants and their fossil analogues. *Plant Ecol* 182:209–233.

17 Pandey KK (1999) A study of chemical structure of soft and hardwood and wood polymers by FTIR spectroscopy. *J Appl Polym Sci* 71:1969–1975.

18 Allard B, Templier J (2000) Comparison of neutral lipid profile of various trilaminar outer cell wall (TLS)-containing microalgae with emphasis on algaenan occurrence. *Phytochemistry* 54:369–380.

19 Bubert H, Lambert J, Steuernagel S, Ahlers F, Wiermann R (2002) Continuous decomposition of sporopollenin from pollen of *Typha angustifolia* L. by acidic methanolysis. *Z Für Naturforschung C* 57:1035–1041.

20 Al Sagheer FA, Al-Sughayer MA, Muslim S, Elsabee MZ (2009) Extraction and characterization of chitin and chitosan from marine sources in Arabian Gulf. *Carbohydr Polym* 77:410–419.
